# Comparative Insights Into the Complete Genome Sequence of Highly Metal Resistant *Cupriavidus metallidurans* Strain BS1 Isolated From a Gold–Copper Mine

**DOI:** 10.3389/fmicb.2020.00047

**Published:** 2020-02-07

**Authors:** Sohaib H. Mazhar, Martin Herzberg, Ibtissem Ben Fekih, Chenkang Zhang, Suleiman Kehinde Bello, Yuan Ping Li, Junming Su, Junqiang Xu, Renwei Feng, Shungui Zhou, Christopher Rensing

**Affiliations:** ^1^Fujian Provincial Key Laboratory of Soil Environmental Health and Regulation, College of Resources and Environment, Fujian Agriculture and Forestry University, Fuzhou, China; ^2^Institute of Environmental Microbiology, College of Resources and Environment, Fujian Agriculture and Forestry University, Fuzhou, China; ^3^Molecular Microbiology, Institute for Biology/Microbiology, Martin-Luther-University Halle-Wittenberg, Halle (Saale), Germany; ^4^College of Plant Protection, Fujian Agriculture and Forestry University, Fuzhou, China

**Keywords:** *Cupriavidus metallidurans*, gold–copper mine, heavy metal efflux systems, cation diffusion facilitators, complete genome

## Abstract

The highly heavy metal resistant strain *Cupriavidus metallidurans* BS1 was isolated from the Zijin gold–copper mine in China. This was of particular interest since the extensively studied, closely related strain, *C. metallidurans* CH34 was shown to not be only highly heavy metal resistant but also able to reduce metal complexes and biomineralizing them into metallic nanoparticles including gold nanoparticles. After isolation, *C. metallidurans* BS1 was characterized and complete genome sequenced using PacBio and compared to CH34. Many heavy metal resistance determinants were identified and shown to have wide-ranging similarities to those of CH34. However, both BS1 and CH34 displayed extensive genome plasticity, probably responsible for significant differences between those strains. BS1 was shown to contain three prophages, not present in CH34, that appear intact and might be responsible for shifting major heavy metal resistance determinants from plasmid to chromid (CHR2) in *C. metallidurans* BS1. Surprisingly, the single plasmid – pBS1 (364.4 kbp) of BS1 contains only a single heavy metal resistance determinant, the *czc* determinant representing RND-type efflux system conferring resistance to cobalt, zinc and cadmium, shown here to be highly similar to that determinant located on pMOL30 in *C. metallidurans* CH34. However, in BS1 another homologous *czc* determinant was identified on the chromid, most similar to the *czc* determinant from pMOL30 in CH34. Other heavy metal resistance determinants such as *cnr* and *chr* determinants, located on megaplasmid pMOL28 in CH34, were shown to be adjacent to the *czc* determinant on chromid (CHR2) in BS1. Additionally, other heavy metal resistance determinants such as *pbr*, *cop*, *sil*, and *ars* were located on the chromid (CHR2) and not on pBS1 in BS1. A diverse range of genomic rearrangements occurred in this strain, isolated from a habitat of constant exposure to high concentrations of copper, gold and other heavy metals. In contrast, the megaplasmid in BS1 contains mostly genes encoding unknown functions, thus might be more of an evolutionary playground where useful genes could be acquired by horizontal gene transfer and possibly reshuffled to help *C. metallidurans* BS1 withstand the intense pressure of extreme concentrations of heavy metals in its environment.

## Introduction

*Cupriavidus metallidurans* BS1 was isolated from a gold copper mine in China and was found to have 99.9–100% 16S rRNA gene identity to available genomes in the National Center for Biotechnology Information (NCBI) database of *C. metallidurans* strains (CH34, NBRC 102507, NDB3NO24, Ni-2, NA1, NA4, NE12 and H1130) using the Geneious alignment (global alignment; cost matrix, identity) (Kearse et al., 2012). The complete genome of *C. metallidurans* BS1 was sequenced because it might give additional insights into the interplay of gold (Au) and copper (Cu) handling in gold biomineralization. *C. metallidurans* CH34 was shown to accumulate toxic Au(I/III) complexes from solution which in turn induced Cu resistance gene clusters, to probably promote cellular defense against oxidative stress (Nies, 1999; Wiesemann et al., 2013). Au(III)-complexes are rapidly reduced to intermediate Au(I)-species by *C. metallidurans* cells and Au(I)-species are subsequently imported into the bacterial cytoplasm (Wiesemann et al., 2013; Zammit et al., 2016) where they exert toxic effects (Jian et al., 2009; Reith et al., 2009).

The strain *C. metallidurans* CH34 was shown to be a facultative chemolitho-autotrophic β-proteobacterium belonging to the family Burkholderiaceae/order Burkholderiales and has been found to be highly resistant to Zn^2+^, Cd^2+^, Ni^2+^, AsO_4_^3–^ CrO_4_^2–^, Hg^2+^, Ag^+^, Cu^1+/2+^, Pb^2+^, and Co^2+^ (Vandamme and Coenye, 2004; Monchy et al., 2007; Janssen et al., 2010). *C. metallidurans* strains are Gram negative and aerobic bacteria that are able to grow on different nutrient sources such as Tris gluconate minimal media, blood agar, tryptic soy agar, and R2A nutrient mediums (Mergeay et al., 1985; Diels and Mergeay, 1990; Gent et al., 2001). Different strains of these bacteria have evolved novel strategies and mechanisms to survive metal-stressed conditions via the exchange of genetic materials through transposons and other mobile genetic elements. *Cupriavidus metallidurans* was shown to survive in various mesophilic metal-contaminated environments such as mining sites for gold, copper and zinc because it possesses a an abundance of transition metal efflux systems and other heavy metal resistance determinants (Diels and Mergeay, 1990; Mergeay et al., 2003). The environmental impacts of mining range from ecosystems destruction with accompanying loss of bio-diversity resources to the accumulation of heavy metals and other pollutants (Mergeay et al., 2003). Gold-rich environments, such as gold mining sites, weathering Au deposits, auriferous soils and sediments embody highly toxic mobile Au-complexes as well as a challenging assortment of transition metals (Mergeay et al., 2003; Rea et al., 2016; Wiesemann et al., 2017).

In *C. metallidurans* CH34, 12 metal-transporting RND (Resistance-Nodulation-Cell division) systems of the heavy metal efflux RND (HME) protein family are known (Tseng et al., 1999; Nies et al., 2006). The plasmid-encoded cobalt–zinc–cadmium resistance pumps CzcCBA and the cobalt–nickel pump CnrCBA have been extensively studied and shown to confer resistance to Co, Zn, Cd, and Ni (Mergeay et al., 1985; Nies et al., 1987, 1989; Collard et al., 1993; Nies, 1995). The *cnr* operon has been reported to have the ability to mutate to additional zinc resistance. There exist extensive homologies in the *cnr* and *czc* encoded structural proteins and the ranges of the exported cations suggest that the two operons have evolved from a common ancestor operon (Collard et al., 1993). This could be due to the ability of both operons to encode determinants for cobalt efflux. CzcD, an important member of the CDF (Cation Diffusion Facilitators) protein family, is part of the *czc* resistance system that transports Zn^2+^, Cd^2+^, and Co^2+^ (Nies, 1992; Anton et al., 2004). Despite the presence of CzcCBA efflux complex, the absence of CzcD decreased cobalt resistance and leads to an extreme sensitive cobalt phenotype when the second CDF protein, DmeF, is also absent (Munkelt et al., 2004; Scherer and Nies, 2009). Hence, this study is targeted at highlighting the genetic rearrangements involved in heavy metal resistance and metals detoxification systems found in a new strain *C. metallidurans* BS1, isolated from a gold–copper mine in China and compare it with the well-studied strain *C. metallidurans* CH34.

## Materials and Methods

### Bacterial Isolation

The heavy metal resistant bacterium, *C. metallidurans* BS1 was isolated from soil samples collected at the mid altitude (590 m) of the gold–copper mine located at the geography coordinates N25° 11.027′ and E116° 24.414′. The bacterium was isolated by inoculating the collected soil samples into Reasoner’s 2A (R2A) agar medium (Qingdao Hope Bio-Technology Co. Ltd., Qingdao, China). There were three replicates of each sample and prior to inoculation of the soil samples on the R2A medium, 1 g of each sample, was mixed with 1000 μL double distilled water (ddw) and incubated for 30 min at 30°C with 150 revolutions per minute (rpm). After incubation, 100 μL of the supernatant was used for the inoculation in R2A medium. The broth culture was incubated at 30°C with shaking at 250 rpm while the agar plates were incubated in the dark at 30°C overnight. The bacterial strain was purified by continuously growing the strain on R2A agar solid plates, without any heavy metal addition, until single colonies were obtained. Finally, the isolates were stored at −80°C in 30% sterile glycerol for long term storage and in −20°C in 30% sterile glycerol for continuous laboratory experiments.

### Minimal Inhibitory Concentration (MIC)

The single colonies were later used to examine the minimal inhibitory concentration (MIC) on CuSO_4_, ZnSO_4_ 7 × H_2_O, CdCl_2_ 5 × 2H_2_O, NaAsO_2_, NiCl_2_ × 6H_2_O, HAuCl_4_ 4 × H_2_O, CoCl_2_ × 6H_2_O and Pb(NO_3_)_2_ supplemented mineral salts media 284 (gluconate 2g/L as sole carbon source) cultures plates. Mineral salts medium 284 contained (1000 ml of DD water) Tris 6.06 g pH 7.0, NaCl 4.65 g, KCl 1.49 g, NH_4_Cl 1.07 g, Na_2_SO_4_ 0.43 g, MgCl_2_ 6^∗^H_2_O 0.20 g, CaCl_2_ 2H_2_O 0.03 g, Na_2_HPO_4_ 2H_2_O 0.23 g, Fe(III)NH_4_ citrate 0.005 g, sodium gluconate 2 g, 1 ml of the trace element solution SL 7 and Difco BactoAgar 15 g L^–1^ (Nies et al., 2006). Medium 284 is a Tris-buffered and low phosphate medium designed to reduce metal complexation and determine accurate MICs (Mergeay et al., 1985).

### Growth Conditions, Genomic DNA Preparation and Molecular Identification

The bacterium strain was grown aerobically in 40 ml R2A broth culture incubated at 30°C with shaking at 250 rpm. After 24 h of growth, the genomic DNA of the bacterium strain was extracted using the TIANamp Bacteria DNA Isolation Kit following the standard protocol provided by the manufacturer (TianGen Biotech, Beijing Co., Ltd.). The quantity and purity of genomic DNA were assessed using an UV spectrophotometry (Nanodrop ND-1000, J & H Technology Co., Ltd.). The OD260/280 value of the genomic DNA higher than 1.80 was picked to check the intact ones via agarose gel electrophoresis (0.8%). Samples containing greater than 25 μg of intact genomic DNA were sent out to perform complete genome sequencing. Molecular identification of the strain was carried out by partial sequencing of the 16S rRNA gene. The 27F/1492R primer pair was used to amplify 16S sequence and the PCR product was subsequently sequenced. Amplification of the 16S rRNA gene was performed using the polymerase chain reaction (PCR). BIO-RAD S1000TM Thermal Cycler (Foster City, CA, United States) machine was used for the PCR and PCR cycle used for amplification was as follows: 5 min at 95°C, followed by 30 cycles of 30 s at 95°C, 30 s at 54°C, 1 min at 72°C and a final extension of 7 min at 72°C. The machine was finally set at 16°C for 2 min for final cooling before the storage of the amplified products. The PCR products were sequenced by Biosune Company (Shanghai, China) using the Sanger method. API^®^ZYM system test kit (bioMérieux, Inc. SA- 69280 Marcy-l’Etoile, France) was used for biochemical characterization of *C. metallidurans* strain BS1 according to manufacturer’s protocol.

The *C. metallidurans* strain BS1 genome was sequenced using a PacBio RS II platform and Illumina HiSeq 4000 platform at the Beijing Genomics Institute (BGI, Shenzhen, China). Four SMRT cells Zero-Mode Waveguide arrays of sequencing, were used by the PacBio platform to generate the sub reads set. PacBio sub reads (length < 1 kb) were removed. The detailed methods of library construction and sequencing can be found at Illumina’s official website^1^. The program Pbdagcon was used for self-correction^2^. Draft genome contigs, which are uncontested groups of fragments, were assembled using the Celera Assembler against a high quality corrected circular consensus sequence subreads set. To improve the accuracy of the genome sequences, GATK^3^ and SOAP tool packages (SOAP2, SOAPsnp, SOAPindel) were used to make single-base corrections. To trace the presence of any plasmid, the filtered Illumina reads were mapped using SOAP to the bacterial plasmid database^4^ (last accessed July 8, 2016).

### Phylogenetic Analysis

Close relative and phylogenetic affiliation of the obtained 16S rRNA sequences were determined by using the BLAST search program at the NCBI website^5^. The 16S rRNA gene sequences were submitted for comparison and identification to the GenBank databases using the NCBI Blastn algorithm and to the EMBL databases using the Fasta algorithm. The phylogenetic tree of 16S rRNA gene sequences and the amino acid sequences of compared resistance determinants were inferred by Geneious prime 2020 0.4. (Geneious Tree Builder) Geneious prime 2020 0.4. (Geneious Tree Builder)^6^, global alignment, gap open penalty 12, gap extension penalty 3, Blosum62 cost matrix, Jukes-Cantor, Neighbor-Joining, Resampling Method – Bootstrap; with 100 replicates). Morphological and physiological experiments were done to further characterize the bacteria.

### Genome Annotation and Characterization

The complete genome sequence of *C. metallidurans* BS1 was submitted to NCBI Prokaryotic Genome Annotation Pipeline (Annotation Software revision 4.6) and The RAST Server: Rapid Annotation using Subsystem Technology (version 2.0) for gene identification following the standard operating procedures. The genes were predicted using Glimmer 3.02 (Delcher et al., 2007) as part of the RAST annotation pipeline (Overbeek et al., 2014). EggNOG 4.5.1 server was used to get the information about genes associated with COG for each predicted protein (Huerta-Cepas et al., 2016), PFAM domains were identified using PFAM 31.0 (Finn et al., 2016). Signal peptides were identified using the SignalP server 4.1 (Petersen et al., 2011), transmembrane helices were inferred using the TMHMM server v. 2.0 (Krogh et al., 2001) and CRISPR repeats were analyzed using CRISPRFinder (Grissa et al., 2007). RAST was used to identify functional genes of interest involved in heavy metal efflux, diffusion and binding systems. Circos software was used to display the circular representation of three replicons of genome of *C. metallidurans* BS1.

### Nucleotide Sequence Accession Number

The complete genome was submitted to NCBI and was released on 21st of March, 2019 with GenBank assembly accession number GCA_003260185.2, under BioProject: PRJNA224116 and BioSample: SAMN08974431.

## Results and Discussion

### Morphology, Growth and Physiology

**FIGURE 1:**
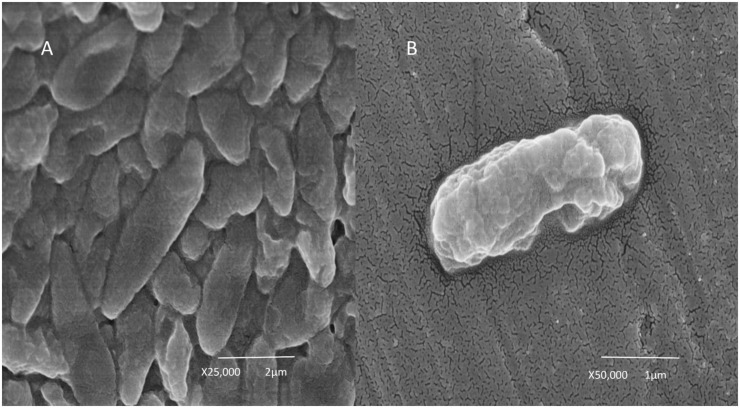
Scanning electron microscopy image of *Cupriavidus metallidurans* BS1. **(A)** Combined growths of bacterial cells and **(B)** Single bacterial cell growth.

**FIGURE 2:**
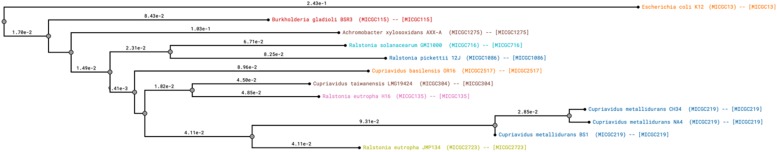
Phylogram - Genome clustering (Microscope) using Mash (Pairwise Genome Distance) and ANI (Average Nucleotide Identity) of *Cupriavidus metallidurans* strains CH34, BS1 and NA4. Names of the analyzed species and the MICGC clusters (MicroScope Genome Cluster) are displayed. A progenome species cluster is defined by computed Mash distances below 0.06 (i.e., ANI > 94%). *Cupriavidus metallidurans* CH34, NA4 and BS1 cluster together in MICGC219 (CH34 _ BS1 = 0.030; CH34 _ NA4 = 0.014) (Konstantinidis and Tiedje, 2005; Blondel et al., 2008; Ondov et al., 2016).

**FIGURE 3:**
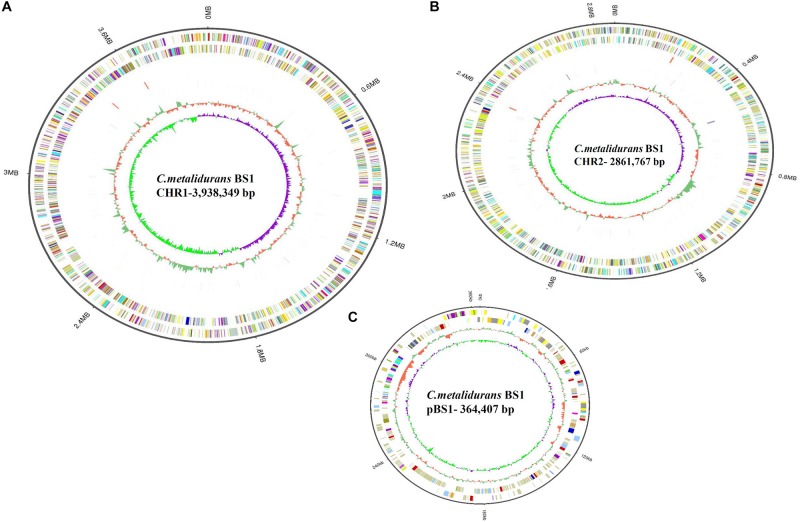
Circular representation of the three replicons of *C. metallidurans* BS1. (**A** – Chromosome, CHR1); (**B** – Chromid, CHR2); (**C** – Plasmid). Circles display from the outwards to inwards, (ring 1): Genome size in MB; (ring 2): Forward strand gene, COG classification; (ring 3): Reverse strand gene, COG classification; (ring 4): Forward strand ncRNA; (ring 5): Reverse strand ncRNA; (ring 6): repeat; (ring 7): GC-content; circle 8: GC-skew (G-C/G + C ratio).

*Cupriavidus metallidurans* BS1 is a Gram negative, motile bacterium in the form of short rods (Figure 1) very similar to the description by Mergeay et al. (1985) about *C. metallidurans* CH34. The colonies of *C. metallidurans* BS1 were creamy in color when grown on R2A agar plates and were also found to exhibit some enzyme activities such as alkaline phosphatase, esterase, esterase lipase, leucine aryl amidase, acid phosphatase and Naphthol-AS-BI-phosphohydrolase. *C. metallidurans* BS1 is free living and can resist high concentration of heavy metals in its natural environment. Species of *C. metallidurans* are reported to be group of short rods (0.8 × 1.2–2.2 μm) bacteria that are oxidase- and catalase-positive, produce indole from tryptophan, assimilate histidine, D-gluconate, adipate and L-malate but not D-glucose, L-arabinose, D-fructose, D-mannose, D-mannitol, *N*-Acetyl-D-glucosamine or maltose as C-source (Mergeay, 2015). The 16S rRNA sequence analysis of BS1 from complete genome assembly showed close relatedness (99.9 – 100% of 1,533 bp) to other strains of *C. metallidurans*, displayed in the phylogenetic tree (Supplementary Figure S1). Phylogram constructed from Genome clustering (Microscope) using Mash (Pairwise Genome Distance) and ANI (Average Nucleotide Identity) represented the high similarities between *C. metallidurans* strains CH34, BS1 and NA4 in Figure 2. Circular representation of the three replicons of genome of *C. metallidurans* BS1 using ncRNA, repetitive sequences, methylation, GC content, GC skew and other information on the genome. GC skew analysis was performed using (G - C)/(G + C) calculations based on Genomic sequences of *C. metallidurans* BS1, the results of gene distribution, ncRNA distribution and gene annotation are also shown on this Figure 3 at the same time. However, at genome level, strain NA4 shows a higher degree of kinship to type strain CH34 than strain BS1. This could be an indication of a genetic drift correlating with global distribution and habitat adaptation (Mergeay et al., 1978; Ali et al., 2019). *C. metallidurans* BS1 conferred resistance to Zn^2+^ displaying a MIC of 20 mM, Cd^2+^ (2.5 mM), Co^2+^ (20mM), Ni^2+^ (8 mM), As^3+^ (3.5 mM), Cu^2+^ (5 mM), Au^3+^ (1 μM) and Pb^2+^ (1.7 mM) on Mineral salts medium 284 plates. The MICs were compared to those obtained with *C. metallidurans* CH34: Zn^2+^ (25 mM), Cd^2+^ (3.5 mM), Co^2+^ (35mM), Ni^2+^ (7 mM), As^3+^ (2.5 mM), Cu^2+^ (3 mM), Au^3+^ (0.5 μM) and Pb^2+^ (1.6 mM) respectively (Table 1). The MICs for zinc, cobalt and cadmium was higher in *C. metallidurans* CH34 while the MICs for nickel, arsenite and copper was higher in *C. metallidurans* BS1 than in *C. metallidurans* CH34. In *C. metallidurans* BS1, *nccCB”B’A nreB* ***mmrQ*** displayed 100% amino acid (A.A) similarity to *C. metallidurans* CH34 but the *cnr* determinants were significantly different (Supplementary Table S6). For example, the nickel diffusion facilitator *cnr*T in *C. metallidurans* BS1 was found having 100% A. A similarity with *Cupriavidus nantongensis* X1 (Fang et al., 2016). The different *cnr* operon might be the reason of the higher MIC for nickel in *C. metallidurans* BS1 compared to *C. metallidurans* CH34.

**TABLE 1:** Minimal inhibitory concentration (MIC) of selected heavy metals for *Cupriavidus metallidurans* BS1 and Cupriavidus metallidurans CH34 in mineral salts media.

	**Zn^2+^ (mM)**	**Co^2+^ (mM)**	**Cd^2+^ (mM)**	**As^3+^ (mM)**	**Ni^2+^ (mM)**	**Cu^2+^ (mM)**	**Au^3+^ (μM)**	**Pb^2+^ (mM)**
BS1	20	20	2.5	3.5	8	5	1	1.7
CH34	25	35	3.5	2.5	7	3	0.5	1.6

### Comparative Genome Annotation and Properties

**FIGURE 4:**
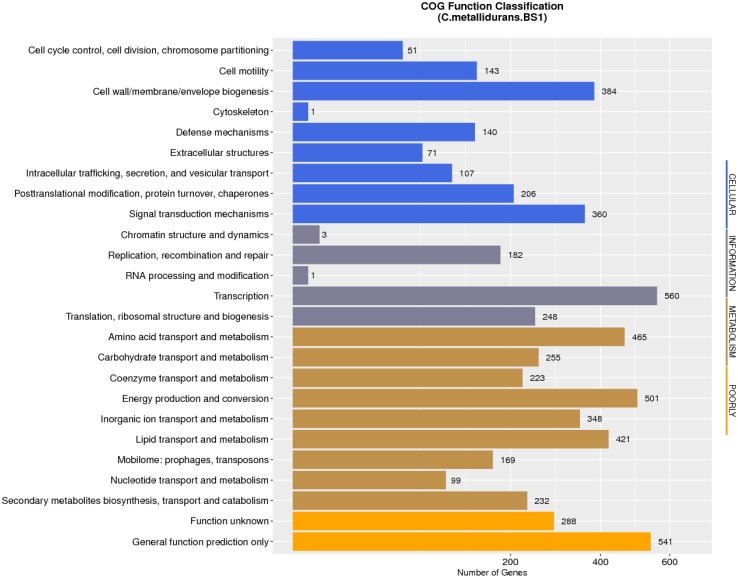
Number of genes associated with general COG functional categories of C. metallidurans strain BS1. COG: Cluster of Orthologous Groups of proteins. This protein database is created and maintained by NCBI. The database is based on the evolutionary relationships of protein systems between bacteria, algae and eukaryotes. Protein sequence are classified into one kind of COG part and each kind of COG part is composed of homologs sequences which are used to deduce the function of the protein. The clusters of orthologous groups of proteins (COGs) were accessed and 5,008 (74.15%) CDS of C. metallidurans BS1 were assigned to one or more COG functional classes COG database is divided into twenty parts by their functions. The statistics is listed above.

The complete genome of *C. metallidurans* BS1 was shown to be 7,164,523 bp in total with 3 replicons and 63.5% G + C content, while the genome of the type strain *C. metallidurans* CH34 is comprised of 4 replicons (chromosome + chromid + pMOL28 + pMOL30) with 6,91335 bp of size in total and identical 63.5% of G + C content. *C. metallidurans* BS1 contained one chromosome (CHR1 – 3,938,349 bp), one chromid (CHR2 – 2,580,084 bp) and one megaplasmid – pBS1 (364,407 bp) close in size to the combined two megaplasmids, pMOL28 and pMOL30 (405,179 bp) but missing almost 40,000 bp, present in type strain *C. metallidurans* CH34 (Janssen et al., 2010). Comparative genomic features between BS1, CH34 and NA4 are shown in Table 2 and general genome statistics are given in Table 3. Several genome sequences of different *C. metallidurans* strains have been analyzed and studied further, including *C. metallidurans* H1130 (102 contigs), *C. metallidurans* NA1 (238 contigs), *C. metallidurans* NA4 (chromosome + chromid + pNA4_A-_D), *C. metallidurans* NE12 (87 contigs), *C. metallidurans* NBRC101272 (63 contigs), *C. metallidurans* NDB3NO24 (38 contigs) and *C. metallidurans* Ni-2 (chromosome + pNi-2_1-3) (Janssen et al., 2010; Van Houdt et al., 2012; Monsieurs et al., 2013, 2014; Mergeay, 2015; Ali et al., 2019). The analyzed strains possess the capability to maintain viability over a wide range of metal (Vandamme and Coenye, 2004; Scherer and Nies, 2009; Van Houdt et al., 2018; Lee et al., 2019). Rfam^7^ predicted that both *C. metallidurans* CH34 and *C. metallidurans* BS1 contained four sets of 5S, 16S, 23S r RNA genes. A difference was found as BS1 was shown to contain 22 sRNA and 63 tRNA genes with 1,900 and 4,927 bp respectively, specifying all 20 amino acids, while CH34 contains 62 tRNA and 12 sRNA (Janssen et al., 2010). RNA analysis for *C. metallidurans* BS1 was performed and predicted using RNAmmer software (V:1.2), compared with rRNA database and Rfam database (Lowe and Eddy, 1997; Lagesen et al., 2007; Gardner et al., 2009; Nawrocki et al., 2015; Chan and Lowe, 2019). *C. metallidurans* BS1 was shown to have 6855 coding sequences (CDS) (Table 2) while MaGe system predicted 6717 coding sequences in type strain *C. metallidurans* CH34 out of which 4,518 CDS are being assigned with functions while the rest of the CDS are hypothetical or conserved hypothetical (Janssen et al., 2010). The clusters of orthologous groups of proteins (COGs) were accessed and 5,008 (74.15%) CDS were assigned to one or more COG functional classes, which were then sorted into 25 groups as shown in Figure 4 (Galperin et al., 2015; Makarova et al., 2015). CDS assigned to COG of type strain CH34 are 5,133 predicted, COGnitor using MaGe system of annotation (Tatusov, 2001). COG analysis assigned 560 genes (maximum) to transcription functions and one gene (minimum) to cytoskeleton and also to RNA processing and modification function. Following transcription, 541 genes were assigned to general function prediction, 501 genes to energy production and conversion, and 465 genes to amino acid transport and metabolism. RAST subsystem analysis placed 28% of the protein coding genes into subsystem categories with the largest percentage assigned to amino acids (AA) and derivatives.

**TABLE 2:** General genomic features of *C. metallidurans* BS1 and *C. metallidurans* CH34.

**Genomic contents**	***C. metallidurans* BS 1**	***C. metallidurans* CH34**	***C. metallidurans* NA4**
Chromosome (CHR1)	3,938,349 bp	3,928,089 bp	3,838,195 bp
Chromid (CHR2)	2,861,767 bp	2,580,084 bp	2,776,395 bp
Single Plasmid	364,407 bp	–*	–*
pBS1 (plasmid)	–*	171,459 bp	–*
pMOL28 (plasmid)	–*	233,720 bp	–*
pMOL30 (plasmid)	364,407 bp	–*	–*
pNA4_A	–*	–*	294,575 bp
pNA4_B	–*	–*	227,796 bp
pNA4_C	–*	–*	155,041 bp
pNA4_D	–*	–*	89,606 bp
GC content%	63.5%	63.5%	63.3%
Total size (bp)	7,164,523 bp	6,913,352 bp	7,381,608 bp
Total CDS	6,616	6717	7611
Number of tRNAs	62	62	64
Contigs (replicons)	3	4	6
Total Gene Number	6,696	6297	7632
Clustered Gene	6110	6258	–
Unclustered Gene	643	39	–

*Not available.

**TABLE 3:** Genome statistics of *C. metallidurans* BS1.

**Attribute**	**Value**	**% of Total**
Genome size (bp)	7,164,523	100.00
DNA coding (bp)	6,213,790	86.73^a^
DNA G + C (bp)	4,549,472	63.5^a^
DNA Scaffolds	3	100.00
Total genes	6753	100.00
Protein coding genes	6692	99.1^b^
RNA genes	97	1.44^b^
Pseudo genes	0	0
Genes in internal clusters	–	–
Genes with function prediction	5213	77.9^c^
Genes assigned to COGs	5364	80.1^c^
Genes with PFAM domains	5360	80.1^c^
Genes with signal peptides	2087	31.2^c^
Genes transmembrane helices	1539	23.0^c^
CRISPR repeats	2	100
1st (CRISPR) credible: spacers	4	
2nd (CRISPR) possible: spacers	1	

*^*a*^Relative to genome size. ^*b*^Relative to total number of genes. ^*c*^Relative to protein-coding genes.*

The chromid in both CH34 and BS1 harbors genes specialized in the activities of sulfonate/taurine transport and utilization, sulfite oxidation, polyhydroxy alkanoic acid (PHA) synthesis and conversion, biofilm formation, and exopolysaccharide synthesis squalene/hopene synthesis, tannin degradation (Janssen et al., 2010). The chromosome (CHR1) was shown to contain genes encoding functions related to DNA replication, DNA repair, translation and transcription and protein processing. The chromid (CHR2) of strain BS1 contained genes encoding determinants putatively responsible for an adaptive response comprising of genes encoding functions such as carotenoid biosynthesis - *carX* (Rmet_5644) is 97%, *crtB* (Rmet_4149) is 99%, tolerance and utilization of acetone - *acxR* (Rmet_4104) 97% (*acxABC* (Rmet_4105-7) absent) similar to the chromid of CH34. A translation initiation factor IF-1 *infA2* (Rmet_5176) in BS1 was 100% similar to the one present in CH34.

### Comparative Genome Plasticity

**FIGURE 5:**
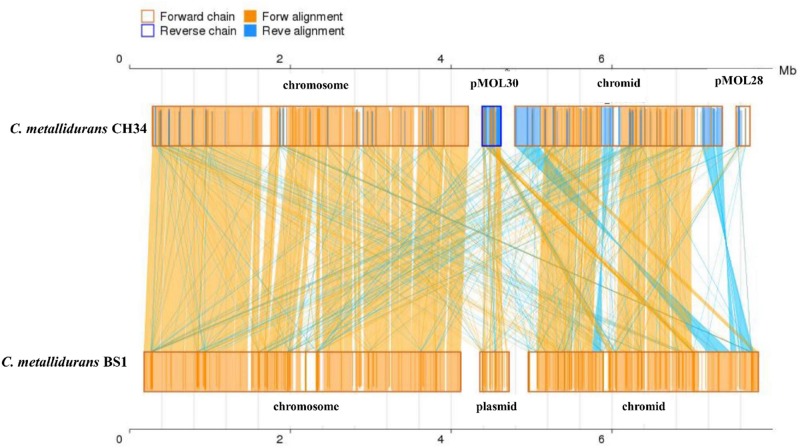
Synteny plot of C. metallidurans strain CH34 and BS1 on nucleic acid level. Synteny was constructed using MCScanX toolkit. The chromosome, chromid and plasmids of both strains were compared. Yellow box represents the forward chain and blue box represents the reverse chain within the upper and following sequence region. In the box of sequence, the yellow region represents the nucleic acid sequence in the forward chain of this genome sequence and the blue region represents the nucleic acid sequence in the reverse chain of this genome sequence. In the middle region of two sequences, the yellow line represents the forward alignment and the blue line represents the reverse complementary alignment.

**FIGURE 6:**
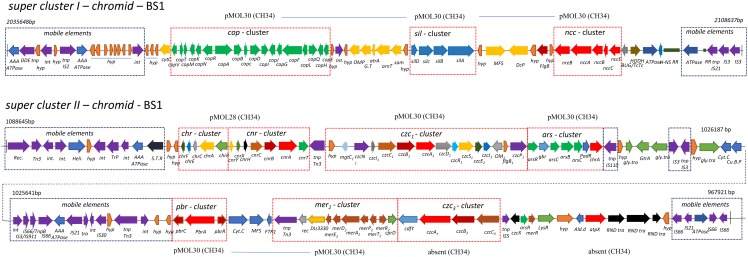
Heavy metals resistance determinant superclusters associated with chromid in *Cupriavidus metallidurans* BS1. Same scale was used for all three lines. Above, the dashed frames are the localization on the corresponding replicon of CH34 as indicated. Super cluster I is comprised of Cop, Sil and Ncc determinants adjacent to each other on chromid between 2,035,648 bp and 2,108,637 bp. Super cluster II includes determinants of Chr–, Cnr–, Czc_1_–, Pbr–, Mer– cluster, CdfY, Czc_3_-cluster and AtpX determinates on chromid between 1,088,645 bp and 967,921 bp. – *int*, integrase; STP, site specific tyrosine recombinase; *hy*p, hypothetical protein; C.H.P, Conserved Hypothetical Protein; R.R, Repeat region; flgB, Flagellar basal-body rod protein; tnp, transposase; P.I, Phage integrase; OMP, Outer Membrane Protein; Glycosyl Transferase; *gtrA*, trA family Protein; *arnT*, Polymyxin resistance protein ArnT; *sam*, SAM dependent methyltransferase; DCP, Diguanylate cyclase/phosphodiesterase domain 2; *SDM*, *S*-adenosylmethionine-dependent methyltransferase; *MSF*, MFS-type transporter; BUG/TcTc, BUG/TctC family periplasmic protein; *HODH*, 2-hydroxy-6-oxo-6-phenylhexa-2,4-dienoate hydrolase; Cyt.C, Cytochrome C; FTR, Iron permease; ald.d, Aldehyde dehydrogenase; CuBP, copper binding protein.

Genome analysis by synteny plot of the *C. metallidurans* strains CH34, BS1 and NA4is showing a high genome plasticity (Figure 5 and Supplementary Figure S2). This genome plasticity is characterized by deletion, insertion and duplication as well as rearrangements on the genomic level caused by horizontal gene transfer, (HGT) transposons, IS elements and prophages. Insertion sequence elements distribution in *C. metallidurans* BS1 was accessed from ISFinder and described in Supplementary Table S1. The BS1 genome showed high similarities to *C. metallidurans* CH34 and other genomes of related *Cupriavidus* strains, particularly related to the chromosome (CHR1) (Millacura et al., 2018). All CMGIs (catabolic-metabolic genomic islands) are missing, probably acquired in *C. metallidurans* CH34 by horizontal gene transfer, on which the genes encoding the hydrogenase, enzymes responsible for the Calvin cycle and metal resistance determinants are also located (Van Houdt et al., 2009) (Table 4). This absence is shown by gaps in the chromosome of strain BS1 (Figure 5). These genomic gaps also included CMGI-2 and CMGI-3, which carry the genes for hydrogen-dependent facultative chemolithotrophy coupled with carbon dioxide fixation (Mergeay et al., 1985; Van Houdt et al., 2009; Herzberg et al., 2015). This leads to a main characteristic in the phenotypic distinction of these two strains. Another missing island is CMGI-1, harboring the gene for an anabolic copper P_IB__1_-type ATPase (CtpA), the gene locus encoding a zinc and cadmium P_IB__2_-type ATPase (CadA) as well as one mercury resistance cluster (Legatzki et al., 2003; Scherer and Nies, 2009; González-Guerrero et al., 2010; Raimunda et al., 2011). Furthermore, the synteny plot shows transfers of several larger gene regions from the pMOL30 and smaller regions of the pMOL28 to the chromid in the BS1 strain. The majority of these regions are loci whose gene products code for metal resistance determinants, especially metal efflux systems. Surprisingly, a large part of these loci were brought into spatial proximity by mobile elements, and two metal-resistance super clusters were formed as newly sorted genome islands (Figure 6 and Supplementary Table S6). The major difference between *C. metallidurans* BS1 and CH34 is that *C. metallidurans* BS1 contained three prophages while it does not have the structural and accessory genes for the soluble and membrane-bound hydrogenase present in *C. metallidurans* CH34 (Mergeay et al., 1985; Janssen et al., 2010; Herzberg et al., 2015). Presence of prophages were also confirmed in *C. metallidurans* NA1, *C. metallidurans* NA4, *C. metallidurans* H1130 and *C. metallidurans* Ni-2 via PHAST^8^ and/or PHASTER (Van Houdt et al., 2018). Strain BS1 contained predicted intact prophages [Bacill_SP_15_ (NC_031245; 7), Burkho_phiE255 (NC_009237; 32), and Ralsto_RSA1 (NC_009382; 29)] as well as NA4, H1130 and Ni-2, detailed information of prophages is mentioned Table 5. Previous studies also provided evidence for the presence of prophages in other strains of *C. metallidurans* than strain CH34 (Van Houdt et al., 2018; Ali et al., 2019).

**TABLE 4:** Comparison and characteristics of the genomic islands of *C. metallidurans* strain CH34 and BS1; (*) (Van Houdt et al., 2012; Ali et al., 2019).

Island (CMGI)*	ORFs (Rmet no._NCBI) (flanking)*		GC%*	length (bp)*	Loci*	**Existence of the islands and flanking genes (CH34/BS1) in BS1 strain**
**Chromosome**			**60.8**			
CMGI-7	0317	0333	61.8	15,398	*arsRIC_2_BC_1_HP*	not existing in BS1 (absent) Rmet_0327 (arsP) = DDF84_RS01615 Rmet_0315 (ssh) = DDF84_RS01605
CMGI-2	1236	1351	63.2	101,635	Hydrogenotrophy, metabolism of aromatic compounds *trbIGFLJEDCB* + organic compont degrading + M-Hyd	not existing in BS1 (absent) Rmet_1235 (helicase) = DDF84_RS06640 Rmet_1352 (bamD) = DDF84_RS06645
CMGI-3	1465	1560	64.0	97,043	CO_2_ fixation and hydrogenotrophy t*rbIGFLJEDXBcopGtraG* + S-Hyd + CO_2_ fixation	not existing in BS1 (absent) Rmet_1463 (guaA) = DDF84_RS07210 Rmet_01565 (FAD) = DDF84_RS07215
CMGI-11	1660	1670	56.4	5,270	Putative fimbrial operon	not existing in BS1 (absent) Rmet_1656 (gshDR) = DDF84_RS07675 Rmet_1671 (phaC) = DDF84_RS07680
CMGI-6		2020	62.5	17,639	No defined function for accessory genes	not existing in BS1 (absent) Rmet_2021 (ACP) = DDF84_RS10755 Rmet_1995 (hyp) = DDF84_RS10745
CMGI-9	2156	2172	56.2	20,610	No defined function for accessory genes	Not existing in BS1 (absent) Rmet_2155 (CytC) = DDF84_RS11470 Rmet_2173 (GGDEF) = DDF84_RS11515
CMGI-1	2287	2408	64.7	109,559	*ctpA* (copper-type ATPase), *merC*_4_*P*_4_*T*_4_*R*_4_ *cdfX*_*pbrR*_2_(*cadR*) < - > *cadA*_pbrC2(*cadC*) *tra*-cluster Almost identical to PAGI-2C of *P. aeruginosa* clone C	Not existing in BS1 (absent) Rmet_2284 (lysR) = DDF84_RS12300 Rmet_2409 (pgA) = DDF84_RS12750
CMGI-8	2549	2561	59.7	12,258	No defined function for accessory genes	Not existing in BS1 (absent) Rmet_2546 (hyp) = DDF84_RS13435 Rmet_2562 (phos) = DDF84_RS13470
CMGI-5	2824	2874	59.6	25,424	Plasmid remnants, Tn6049 inserted in a gene involved in plasmid mobilization relaxase	Not existing in BS1 (absent) Rmet_2823 (hnh) = DDF84_RS14735 Rmet_2849 (M48) = DDF84_RS14745
CMGI-4	2987	3045	62.2	56,530	*parB*_*dpd*_*repA*_ferrric reductase_S_R_*hmzCA*‘_ *phnCBE* No conjugative module,	Not existing in BS1 (absent) Rmet_2986 (endon) = DDF84_RS15430 Rmet_3046 (malic) = DDF84_RS15435
CMGI-10	3347	3368	56.0	20,712	No defined function for accessory genes	Not existing in BS1 (absent) Rmet_3346 (uspA) = DDF84_RS16990 Remt_3369 (paaA) = DDF84_RS16995
CMGI-12	2662	2670	50.9	8,767	No defined function for accessory genes	Not existing in BS1 (absent) Rmet_2671 (yeeE) = DDF84_RS13965 Rmet_2660 (hyp) = DDF84_RS13955
CMGI-13	2723	2737	55.6	15.909	Genes involved in polysaccharide biosynthesis	Not existing in BS1 (absent)
**Chromid**			**63.8**			
CMGI-A	4428	4305	63.2	87,100 (124,200)	No defined function for accessory genes, only Transposon insertin in flanked regions	exist in BS1 and CH34 Rmet_4306 (MFS) = DDF84_RS20955 Rmet_4428 (moaC) = DDF84_RS21810
CMGI-B + D	4475 4598	4496 4715	61.2 63.6	22,080 141,200 = 160,700	Includes *rpoD*_2_, hyd4, *gig*, *rpoQ* CH34-this insertion disrupts the *czcICBA* locus to *czcICB‘-czcB“A*	CMGI-D’ (98,946 bp, Rmet_4598 – Rmet_4687) not existing in BS1 (absent) Rmet_4688 (hyp) = DDF84_RS23310
CMGI-C	4450	4556	64.4	7,100 (6,150)	TBSSR fragment, gene coding for mannose-binding lectin	Exist in BS1 and CH34 Rmet_4450 (plp) = DDF84_RS21915 Rmet_4456 (cupin) = DDF84_RS21945
CMGI-E	5454	5568	57.5	121,400	Tn7-related genes at one extremity, genes putatively involved in degradation of aromatic compounds	Not exist in BS1 (absent) Rmet_5440 (thio) = DDF84_027535 Rmet_5568 (hyp) = DDF84_031170
CMGI_ BS1_1	DDF84 _RS2793	DDF84 _RS28445	62.5	109,796	Flanked by transposons_intergreases; genes for unknown MFS transporter, RND systems and possible different metabolic pathways	Not exist in CH34 (absent) Rmet_3744 (flgL) = DDF84_RS28450 Rmet_5440 (thio) = DDF84_027535 (+CMGI-30b)
**pMOL28**			**60.5**			
CMGI-28a	6212	6333	61.8	42.846	Heavy metal resistance genes (mer, *cnr* and *chr*), flanked by IS*1071* elements	Transfered to chromid in BS1 Rmet_6212 (Tn3) = DDF84_023150 Rmet_6344 (merR1) = DDF84_023300 (merR3) transfered to chromid in BS1 at CMGI-D’ site in CH34; Rmet_4687 = DDF84_023305
CMGI-28b	6252	6263		23.000	TBSSR is part of a four-gene module (PRQ), three *hrs*-like genes of unknown function that code for products rich in tyrosine-aspartate motifs	Not exist in BS1 (absent)
CMGI-28c	6320	6332		15.000	CMGI-28c sited adjacent to CMGI-28a, contains mostly hypothetical genes: their proximity suggests that these two might constitute one single genomic island. BSSR, no defined function for accessory genes	Not exist in BS1 (absent)
**pMOL30**			**60.1**			
CMGI-30a	6002	6171	61.2	74.399	TBSSR, heavy metal resistance genes (*mer*, *czc* and *pbr*) flanked by Tn4380 elements (one intact and one truncated copy)	Remains on pBS1 Rmet_5986 (hyp) = DDF84_023140 Rmet_5966 (gtf) = DDF84_33475 Duplicated and transfered to chromid in BS1 Rmet_5986 (hyp) = DDF84_023140 Rmet_6171 (merR2) = DDF84_022830
CMGI-30b	6153	6067	60.5	87.900	TBSSR, heavy metal resistance genes *(sil*, *ncc*, and *cop*)	CMGI-30b‘ RMET_RS20695 (hyp) = DDF84_027595 RMET_RS30405 (hyp) = DDF84_027910; transfered to chromid in BS1 at CMGI-E site in CH34; DDF84_027575– DDF84_027535

*TBSSR; tyrosine-base site specific recombinase.*

**TABLE 5:** Prophages observed in *C. metallidurans* BS1, NA1, NA4, H1130, and Ni-2.

**Strain replicon**	**Region length (Kb)**	**Completeness^a^**	**Score^b^**	**Total proteins**	**Region position**		**Most common phage^c^**	**G + C%**
**CH34**								
NC_007973	22.6	Incomplete	30	7	1,572,881	1,595,502	Salmonella phage SSU5	61.15
**BS1**								
NZ_CP037900	13.8	Incomplete	20	15	1,282,622	1,296,518	Burkho_phiE125 (NC_003309; 2)	62.01
NZ_CP037900	24.4	Incomplete/intact	30	17	1,287,577	1,311,986	Bacter_APSE_2 (NC_011551; 1)	62.47
NZ_CP037900	20.6	Overlapping	70	18	1,314,007	1,334,670	Bacill_SP_15_ (NC_031245; 7)	62.87
NZ_CP037900	15.9	Incomplete	40	19	1,322,497	1,338,448	Salmon_PVP_SE1 (NC_016071; 2)	63.42
NZ_CP037900	38.2	Intact	100	52	1,849,213	1,887,418	Burkho_phiE255 (NC_009237; 32)	62.75
NZ_CP037900	43.2	Intact	110	55	2,186,334	2,229,622	Ralsto_RSA1 (NC_009382; 29)	60.79
NZ_CP037900	30	Questionable	70	24	2,589,667	2,619,673	Rhodof_P26218 (NC_029061; 8)	58.73
NZ_CP037900	19	Incomplete	30	11	2,878,551	2,897,641	Acinet_vB_AbaS_TRS1 (NC_031098; 3)	62.74
NZ_CP037901	22.3	Incomplete	60	12	925,371	947,671	Entero_BP_4795 (NC_004813; 3)	56.8
NZ_CP037901	11.2	Questionable	90	12	1,014,984	1,026,217	Entero_fiAA91_ss (NC_022750; 2)	61.07
NZ_CP037902	7.5	Incomplete	50	12	176,984	184,518	Entero_BP_4795 (NC_004813; 3)	59.8
NZ_CP037902	9.6	Incomplete	20	11	275,695	285,333	Entero_N15 (NC_001901; 2)	60.24
NA1*	27.9	Questionable	90	32	528,474	556,451	Ralsto_RS138 (NC_029107; 7)	65.35
	17.7	Incomplete	20	21	554,542	572,263	Pseudo_NP1 (NC_031058; 3)	64.23
NA4*	43.6	Intact	100	50	1,706,628	1,750,233	Bordet_BPP_1 (NC_005357; 18)	64.94
	6.1	Intact	100	10	1,941,664	1,947,835	Ralsto_PE226 (NC_015297; 6)	60.08
	45.2	Intact	150	41	2,145,854	2,191,126	Burkho_Bcep176 (NC_007497; 11)	61.83
	8	Incomplete	30	10	2,181,367	2,189,450	Gordon_Nymphadora (NC_031061; 2)	62.44
	120.5	Intact	130	125	2,248,504	2,369,042	Salmon_118970_sal3 (NC_031940; 14)	61.84
	44.5	Incomplete	30	39	2,545,435	2,589,959	Pseudo_JBD44 (NC_030929; 5)	63.89
H1130*	19.3	Incomplete	30	21	1,470,543	1,489,908	Burkho_phiE125 (NC_003309; 3)	61.27
	12.7	Incomplete	40	19	1,505,965	1,518,710	Bacill_SP_15 (NC_031245; 5)	63.39
	7.9	Incomplete	30	9	2,763,200	2,771,108	Entero_phi92 (NC_023693; 4)	58.60
	48.4	Intact	110	73	6,748,679	6,797,173	Salmon_SEN34 (NC_028699; 22)	62.09
	16	Incomplete	50	29	7,110,429	7,126,525	Clostr_phiCT453B (NC_029004; 4)	61.04
**Ni-2**								
NZ_CP026544	15.9	Incomplete	10	19	820,378	836,317	Ralsto_RSM3 (NC_011399;6)	59.25
NZ_CP026544	44.8	Intact	150	64	998,191	1,043,088	Salmon_64795_sal3 (NC_031918; 13)	62.59
NZ_CP026544	21.6	Incomplete	20	15	393,007	1,414,698	Bacill_Stahl (NC_028856; 3)	61.63
NZ_CP026544	17.2	Incomplete	30	23	1,402,983	1,420,235	Acinet_LZ35 (NC_031117; 5)	62.93
NZ_CP026544	20.2	Questionable	90	24	1,428,082	1,448,374	Pseudo_JBD44 (NC_030929; 4)	62.85
NZ_CP026544	14.5	Incomplete	60	11	4,491,466	4,506,010	Salmon_SJ46 (NC_031129; 3)	62.43
NZ_CP026546	6.5	Incomplete	10	11	4,219	10,807	Entero_N15 (NC_001901; 2)	59.99

*No complete Sequence available for sorting to replicons (Van Houdt et al., 2018). ^*a*^Prediction of whether the region contains an intact or incomplete prophage, ^*b*^score based on PHASTER criteria (Zhou et al., 2011), ^*c*^the phage with the highest number of proteins most similar to those in the region (between parentheses: accession number; number of proteins).

### Determinants of Metal Homeostasis and Genomic Resistance Superclusters

**FIGURE 7:**
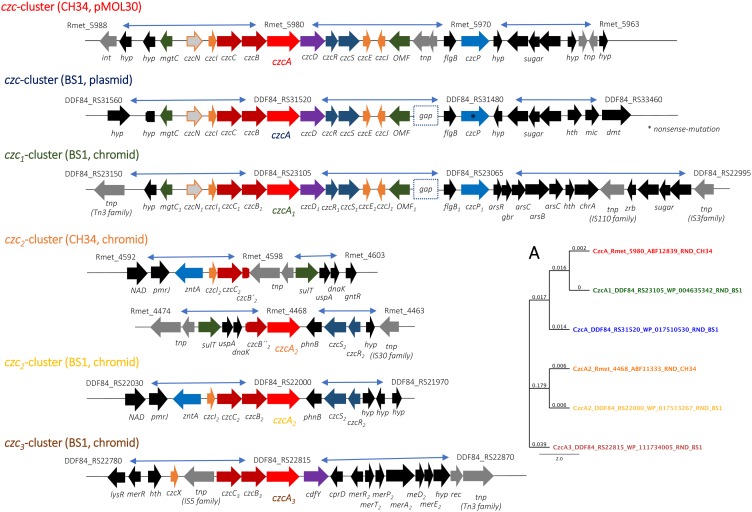
Comparison of the *czc* cluster - synteny of the *C. metallidurans* strains BS1 and CH34. Shown are the loci of the *czc* cluster of *C. metallidurans* CH34 on pMOL30 and the chromid as well as of BS1 on the plasmid and chromid, as well as the synteny of these gene regions. In bright red and highlighted are *czcA* genes as well as in dark red the structural genes of the tripartite RND efflux system, in orange genes encoding for associated periplasmic resistance proteins, in violet genes coding for CDF proteins, in blue genes encoding P-type ATPases, in dark green genes encoding transporters of the outer membrane, in gray genes encoding transposases, dark blue genes encoding the associated 2-component systems and in black genes encoding putative functions of the flanking region. *Int*, integrase; hyp, hypothetical gene; *tnp*, transposase; OMF, outer membrane factor; *hth*, helix-turn-helix transcriptional regulator; *mic*, mechanosensitive ion channel family protein; *dmt*, DMT family transporter; *gbr*, glyoxalase/bleomycin resistance/dioxygenase family protein; *zrb*, zinc ribbon domain-containing protein; sugar, glycosyltransferase family 2 protein, *GtrA* family protein, glycosyl transferase; NAD, NAD-dependent dehydratase; rec, recombinase family; *CprD*, cupredoxin like protein**. (A)** Molecular Phylogenetic tree based on the full amino acid sequences of CzcA encoded on pMOL30 of *C. metallidurans* CH34 and the homologues as well as paralogous CzcA (HME-RND) proteins of both strains. In addition to the gene names, the respective locus tags and NCBI GenBank protein ID numbers of both strains are indicated. In addition, in color matching the synteny representation was marked. The evolutionary history was inferred by using the Geneious prime 2019.2.1 (Geneious Tree Builder) (https://www.geneious.com), global alignment, gap open penalty 12, gap extension penalty 3, Blosum62 cost matrix, Jukes-Cantor, Neighbor-Joining. The tree was drawn to scale, with branch lengths calculated using the average pathway method; the scale bar corresponds to the number of substitutions per site (Kearse et al., 2012).

In order to maintain metal homeostasis in the presence of multiple heavy metals, the transportome contains the metal uptake systems in addition to the metal efflux systems as the first pillar of defense (Nies, 2016). The strain BS1 shows a high degree of conservation on gene and amino acid sequence level as well as the gene loci synteny of the metal uptake systems encoding genome regions in comparison to strain CH34. In contrast to the transportome, the knowledge about the direct interaction with the metal handling proteins of the cytoplasm, cytoplasmic membrane and the periplasm, which form the metal repositories as the second pillar of bacterial metal homeostasis (Herzberg et al., 2014b), is underrepresented but further investigation is in progress (Herzberg et al., 2014a, 2016; Bütof et al., 2017; Chandrangsu et al., 2019). In order to adapt the cellular metal homeostasis to the specific environmental conditions, its regulation is of central importance as a third pillar. Besides the one- and two-component regulatory systems, sigma factors of the extracytoplasmic function (ECF) family are of high importance to adapt the metal transport and metal handling to the specific cellular requirements and to avoid toxic effects of the various heavy metals (Missiakas and Raina, 1998; Heimann, 2002; Mascher, 2013; Große et al., 2019). The ECFs and ICFs (intracytoplasmic function), as well as the metal uptake systems, show a high degree of similarity on the gene and amino acid sequence level as well as the gene loci synteny. One exception is the ECF CnrH, which was transferred from the plasmid to the chromid with the entire *cnr* cluster by mobile elements and is part of the metal super cluster I structure (Grass et al., 2000). The alternative RNA polymerase sigma-70, ICF – RpoD_2_ (Rmet_4661, on chromid in CH34), and ECF RpoQ (Rmet_4686, on chromid in CH34) are not present on the genome of strain BS1 due to the lack of CMGI-D. Instead of the missing sigma factor, strain BS1 harbors one additional (SigX) with 74.5% similarity on amino acid level to RpoO (Supplementary Table S2). This metallophilic strain BS1, harbors numerous gene clusters encoding metal-resistance determinants enabling detoxification of transition metal ions and complexes (Nies, 2003, 2016). With regard to synteny and genome plasticity, mediated by mobile elements, *C. metallidurans* strain BS1 displayed a transfer of the metal resistance super cluster I from plasmid (pMOL30 in CH34) to chromid (Supplementary Table S4 and Figures 6, 7). The generation of an extended cluster II on chromid could also be observed. In this second cluster, the *chr* - *cnr*, *czc*_1_, *pbr*, *czc*_3_ as well as *cdfY* (encoding an not exist CDF family protein in CH34) and *atpX* (encoding an not existing P-type ATPase family protein in CH34) were brought into spatial proximity on the chromid (Supplementary Tables S5, S6 and Figures 6, 7).

### Comparative Overview of the Encoded Heavy Metal Resistance Systems on the Genomes of *C. metallidurans* BS1 and CH34

The encoded determinants of heavy metal resistance that were found on the genome of BS1 included chemiosmotic efflux of cations with proton antiporters of the HME-RND family (Heavy Metal Efflux – Resistances, Nodulation and Cell Division, TC_2.A.6.3) having three-component cation efflux systems such as a well-studied CzcCBA system (cobalt zinc cadmium), CnrCBA (cobalt nickel), CusCBA (copper, silver), NccCBA (nickel, cobalt and cadmium) and plenty of additional genes encoding cation diffusion facilitators (CDF family, TC_2.A.4) such as *czcD, dmeF and fieF*, a member of the DMT (permeases of the drug/metabolite transporter, TC_2.A.7) family such as *cnrT*, major facilitator superfamily (MFS super family) such as *nccT/nreB* and also cation P-type ATPases for cytoplasmic detoxification (*zntA, pbrA*, *cupA*/*copF*, and *czcP*) (Aziz et al., 2008) detoxifying Cu(I)/Ag(I), Pb(II)/Cd(II)/Zn(II) and Hg(II) ATPases (Silver et al., 1993). Members of the P-type ATPases and CDF protein family transport excess heavy metals such as Zn^2+^, Cd^2+^ or Co^2+^ from the cytoplasm to the periplasm (Silver et al., 1993; Fagan and Saier, 1994; Ashburner et al., 2000; Montanini et al., 2007; Nies, 2016) and the proton-driven efflux systems such as CzcCBA export these metals from the periplasm to the outside of the cell (Paulsen and Saier, 1997; Nies, 2003) as a two stage mode of action to prevent toxic effects by metal excess. The genes encoding *chr* (Cr), *czc* (Co, Zn, Cd) and *ncc* (Ni, Co, Cd) in *C. metallidurans* BS1 could be identified with up to 100% AA sequence similarities to those found in *C. metallidurans* CH34. This high number of different resistance determinants enable multiple resistance mediation and ability to handle the following metals; Zn^2+^, Cd^2+^, Ni^2+^, Ag^+^, Cu^1+/2+^, Pb^2+^, and Co^2+^, from the micromolar to the millimolar concentration range.

### P-Type ATPases

**FIGURE 8:**
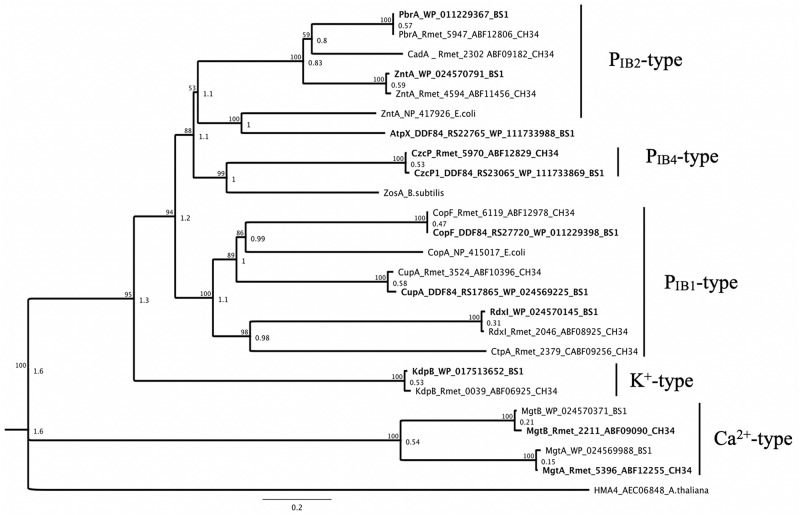
Molecular Phylogenetic tree based on the full amino acid sequences of P-type ATPases encoded by the genome of C. metallidurans CH34 and the homologs as well as paralogous of both strains. In addition to the gene names, the respective locus tags and NCBI GenBank protein ID numbers of both strains are indicated, BS1 are bold. The evolutionary history was inferred by using the Geneious prime 2020 0.4. (Geneious Tree Builder) (https://www.geneious.com), global alignment with free gaps, Identity (1.0/0.0), gap open penalty 12, gap extension penalty 3, 2 refinement iterations, Genetic Distance Model _ Jukes-Cantor, Neighbor-Joining, Resampling Method – Bootstrap; 100. The ATPase subfamilies are indicated (Nies, 2016). The ZntA*_*E.coli*_*, ZosA*_*B.subtilis*_*, HME4*_*A.thaliana*_*, and CopA*_*E.coli*_* (P-type ATPase family) amino acid sequences from Escherichia coli strain K-12 substr. MG1655, *Bacillus subtilis* and *Arabidopsis thaliana* were used as reference (Rensing et al., 1997, 2000; Fan and Rosen, 2002; Okkeri and Haltia, 2006; Wang et al., 2014). The tree is drawn to scale, with branch lengths calculated using the average pathway method; the scale bar corresponds to the number of substitutions per site, the branch labels are consensus Support (%) and Node Heights (Kearse et al., 2012).

At the first level of detoxification, the cytoplasmic efflux, the two strains BS1 and CH34 showed a high degree of similarity between the metal-transporting ATPases (Figure 8). In both genomes existed gene loci coding for the P_IB__2__/__4_-type ATPases ZntA, PbrA and CzcP, which are primarily involved in Zinc, cadmium, cobalt and lead efflux, with a 97–100% conservation at the amino acid level (Wang et al., 2014). This is also reflected in the copper-transporting P_IB__1_-type ATPases CupA, CopF, and RdxI, homologous of the *E. coli* CopA (Rensing et al., 2000; Fan and Rosen, 2002; Raimunda et al., 2011; Wiesemann et al., 2013, 2017). Exceptions are found in the non-existent P-type ATPases CadA and CtpA, due to the lack of CMGI-1 in the genome of strain BS1. In contrast, the locus tag DDF84_RS22765 encodes a P-type ATPase (AtpX) not present in strain CH34, which displayed a higher similarity on amino acid-level to ZntA of *E. coli* than to ZntA of BS1 or CH34 (Rensing et al., 1997; Wang et al., 2014). The ATPases involved in the uptake of potassium (K^+^-type) or calcium (Ca^+^-type) are present in both strains with a similarity of 98-99% (Maguire, 2006; Cromie and Groisman, 2010) (Figure 8). The family of MerR regulators includes members involved in the gene regulation of zinc-, cadmium-, cobalt-, lead- and copper-exporting P-type ATPases, of mercury resistance systems, and are involved in the oxidative stress response (Brown et al., 2003; Hobman et al., 2005; Tottey et al., 2005; Molina-Henares et al., 2009; Ibáñez et al., 2015).

### CDF Proteins

**FIGURE 9:**
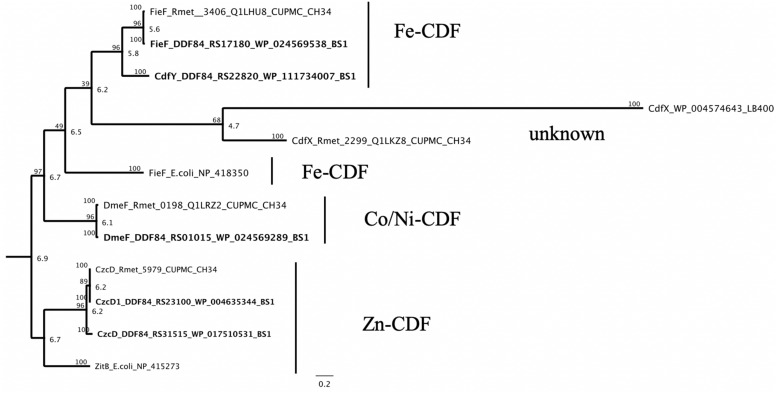
Molecular Phylogenetic tree based on the full amino acid sequences of the CDF (cation diffusion facilitator family) protein encoded on the genome of *C. metallidurans* CH34 and the homologs as well as paralogous of both strains. In addition to the gene names, the respective locus tags and NCBI GenBank protein ID numbers of both strains are indicated, proteins encoded on the genome of BS1 are displayed in bold. The evolutionary history was inferred by using the Geneious prime 2020 0.4. (Geneious Tree Builder) (https://www.geneious.com), global alignment with free gaps, Identity (1.0/0.0), gap open penalty 12, gap extension penalty 3, 2 refinement iterations, Genetic Distance Model _ Jukes-Cantor, Neighbor-Joining, Resampling Method – Bootstrap; 100. The CDF subfamilies are indicated (Montanini et al., 2007). The ZitB*_*E.coli*_*, FieF*_*E.coli*_* and CdfX*_*P.xenovorans*_* (CDF family) amino acid sequences from Escherichia coli strain K-12 substr. MG1655 and Paraburkholderia xenovorans LB400 were used as reference (Lee, 2002; Van Houdt et al., 2009). The tree is drawn to scale, with branch lengths calculated using the average pathway method; the scale bar corresponds to the number of substitutions per site, the branch labels are consensus Support (%) and Node Heights (Kearse et al., 2012).

Genes for the three more closely characterized members of the CDF protein family from *C. metallidur*ans CH34, CzcD, DmeF and FieF, could also be found with a high degree of conservation on the genome of the *C. metallidur*ans BS1 studied here (Scherer and Nies, 2009). The *czcD* gene occured two times (*czcD*/*czcD*_1_) by duplication of the *czc* cluster on plasmid and chromid in BS1 and the plasmid encoded CDF protein showed a lower similarity with 91.5% in comparison to the chromid encoded version with 100% similarity on the amino acid level to CzcD of CH34 (Figure 7 and Supplementary Table S4). CzcD clustered with ZitB of *E. coli* in one separated branch in the phylogenetic tree and could be sorted to the Zn-CDFs (Anton et al., 2004; Montanini et al., 2007). DmeF is mainly involved in the cobalt homeostasis and occured in both strain with 96.6% similarity (Munkelt et al., 2004; Scherer and Nies, 2009). A CdfX (locus tag: Rmet_2299) homolog was absent in strain BS1 due to the lack of CMGI-1 (Table 4). The other homolog to the CdfX is known from *Paraburkholderia xenovorans* LB400 and indicated a common origin and acquisition through horizontal gene transfer (Van Houdt et al., 2009). The Fe/Zn-CDF member FieF (Rmet_3406) encoded on chromosome of *C. metallidurans* CH34 shared an amino acid similarity of 99.7% with his homologous in *C. metallidurans* BS1 and cluster with FieF (YiiP) of *E. coli* as well as with an unknown and uncharacterized CDF (CdfY) member only encoded on chromid in BS1 (Munkelt et al., 2004; Grass et al., 2005; Lu and Fu, 2007) (Figure 9).

### The RND Pumps

**FIGURE 10:**
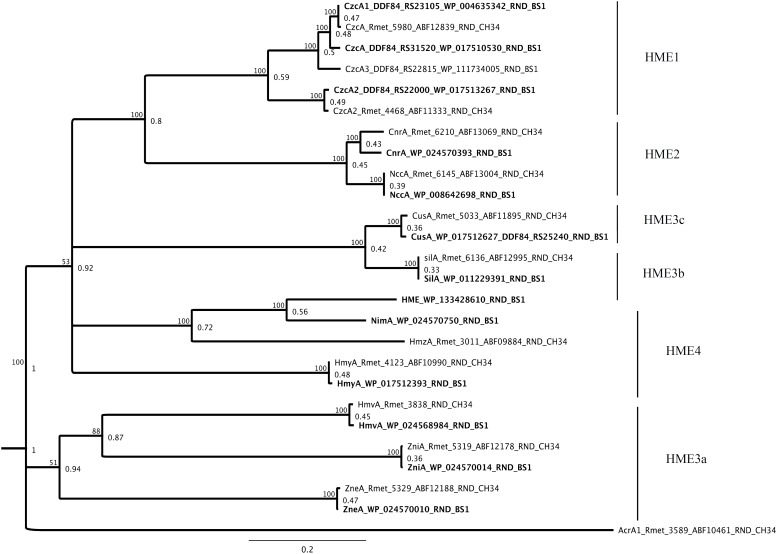
Molecular Phylogenetic tree based on the full amino acid sequences of the HME-RND members encoded on the genome of *C. metallidurans* CH34 and the homologs as well as paralogous of both strains. In addition to the gene names, the respective locus tags and NCBI GenBank protein ID numbers of both strains are indicated. The HME subfamilies are indicated (Nies, 2016). The evolutionary history was inferred by using the Geneious prime 2020 0.4. (Geneious Tree Builder) (https://www.geneious.com), global alignment, gap open penalty 12, gap extension penalty 3, Blosum62 cost matrix, Jukes-Cantor, Neighbor-Joining, Resampling Method – Bootstrap; 100. AcrA1 (Rmet_3589_ABF10461) from CH34 is used as an outgroup as a member of the HAE-RND family. The tree is drawn to scale, with branch lengths calculated using the average pathway method; the scale bar corresponds to the number of substitutions per site, the branch labels are consensus Support (%) and Node Heights (Kearse et al., 2012).

The bacterial tripartite RND systems are very powerful metal efflux pumps, known from the best studied members *czc*, *cnr* and *cus* (Kim et al., 2011; Long et al., 2012; Nikaido, 2018). The presence of genes encoding 14 members associated with MFP (membrane fusion protein) and OMF (outer membrane factor) in synteny was detected and these could be sorted to the HME-RND efflux systems in BS1 which have been shown to be involved in heavy metal resistance/tolerance (Figure 10). In this study, we found homologs of the HME1 [CzcA and HmuA (CzcA_2_)], HME2 (CnrA and NccA), HME3a (ZniA, ZneA, and HmvA), HME3b (NimA and HmzA), HME3c (HmyA) and HME4 (CusA and SilA) group (Nies et al., 2006; Edward et al., 2013; Nies, 2016). The gene loci of the three component efflux systems (Sil, Cnr, Ncc) were encoded on chromid in *C. metallidurans* BS1 while on type strain *C. metallidurans* CH34, they were shown to be present on different plasmids (pMOL28 and pMOL30) (Janssen et al., 2010) (Supplementary Table S6). In addition, genes encoding two-component-regulatory system in *C. metallidurans* BS1 were annotated as “DNA binding heavy metal response regulator,” for CzcR and as “heavy metal sensor histidine kinase” for CzcS. These genes were found adjacent to a gene encoding CzcP, characterized as cation efflux P_1__B__4_-type-ATPase involved in Zn homeostasis and part of *czcNICBADRSJE_czcP* cluster (Nies, 1992; Grosse et al., 2007; Fairbrother et al., 2013).

### Gold–Copper Detoxification Systems of *C. metallidurans* BS1 and CH34

**FIGURE 11:**
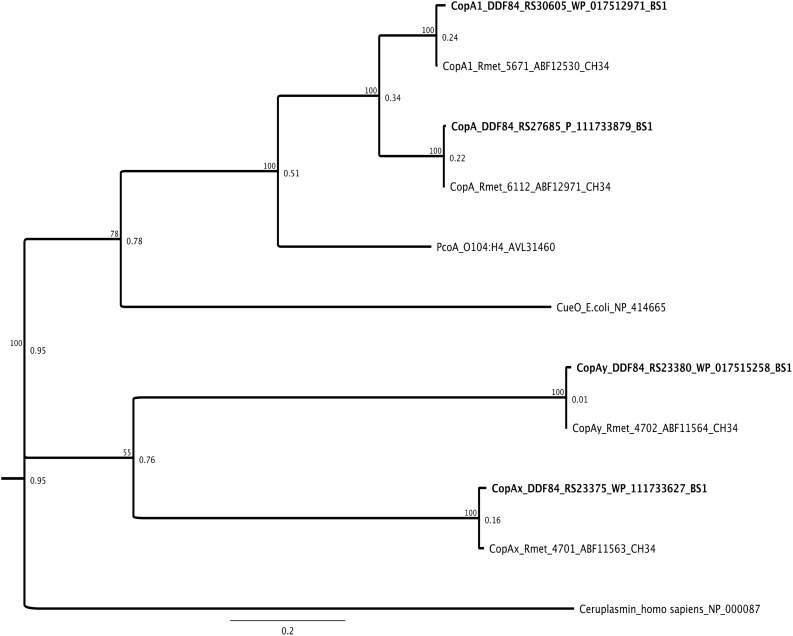
Molecular Phylogenetic tree based on the full amino acid sequences of the CopA (multi copper oxidases) protein encoded on the genome of *C. metallidurans* CH34 and the homologs as well as paralogous of both strains. In addition to the gene names, the respective locus tags and NCBI GenBank protein ID numbers of both strains are indicated, multicopper oxidases from BS1 are displayed in bold. The evolutionary history was inferred by using the Geneious prime 2020 0.4. (Geneious Tree Builder) (https://www.geneious.com), global alignment with free gaps, Identity (1.0/0.0), gap open penalty 12, gap extension penalty 3, 2 refinement iterations, Genetic Distance Model _ Jukes-Cantor, Neighbor-Joining, Resampling Method – Bootstrap; 100. The CueO*_*E.coli*_*, PcoA*_*E.coli*_* and Ceruplasmin (multi copper oxidase family) amino acid sequence from Escherichia coli strain K-12 substr. MG1655 and O104:H4 and Homo sapiens was used as reference. The tree is drawn to scale, with branch lengths calculated using the average pathway method; the scale bar corresponds to the number of substitutions per site, the branch labels are consensus Support (%) and Node Heights (Kearse et al., 2012).

The annotation of *C. metallidurans* BS1 genome revealed a high degree of similarity of metal resistant factors to the well-studied *C. metallidurans* strain CH34. Overall, the genome of this bacterium contained 42 genes coding for proteins indirectly or directly involved in copper homeostasis including Cu resistance proteins encoded by the *copS_1_R_1_A_1_C_1_B_1_D_1_* gene cluster as well as another homolog but extended gene cluster *copHMKNSRACBDIJGFLQHE* (Supplementary Table S3), four multicopper oxidases (CopA, CopA_1_, CopA_X_, and CopA_Y_) (Figure 11), the RND system CusFCBA and SilDCBA (Supplementary Table S6) and three copper-translocating P_IB__1_-type ATPases (CupA, CopF, and RdxI) (Figure 8). The fourth, anabolic, P_IB__1_-type ATPases (CtpA) from the genome of strain CH34 encoded on CMGI-D is not present due to the absence of this genomic island in strain BS1 (Nies, 2016) (Table 4). Data exist for several of these systems that show their direct involvement in copper homeostasis and their induction of gene expression under increasing copper concentrations in strain CH34, including the two *cop* clusters, the divergon *cupCAR* and the *cus* operon (Nies et al., 2006; Wiesemann et al., 2013). Furthermore, parallels are known regarding the induction of these clusters in the comparison between copper and gold. With increasing gold concentrations and, as a result, increased uptake of Au(III) complexes, the induction of the *cop* clusters and *cup* divergon occurs in addition to the induction of *mer* genes (part of the mercury detoxification by reduction and export of Hg(II) (Reith et al., 2009; Rojas et al., 2014). The e MerR-type transcriptional regulator CupR/CueR (Rmet_3523) from *C. metallidurans* was shown to be activated by Au-complexes (Stoyanov and Brown, 2003; Checa et al., 2007; Jian et al., 2009; Wiesemann et al., 2013; Ta et al., 2014) (Supplementary Figure S3) leading to formation of the CupA (P_IB__1_-type ATPase) efflux pump. Many predicted protein homologies were found in both *C. metallidurans* BS1 and the well characterized strain *C. metallidurans* CH34. The *cupR* and *cupA* gene products from *C. metallidurans* CH34 have 99 and 98% amino acid (AA) sequences identity, respectively, to those found on the genome of *C. metallidurans* BS1 (Figure 8) (Altschul, 1997). These genes were located downstream of *cupC*, encoding a putative copper chaperone, which has 100% AA sequence similarity with *cupC* encoded on chromosome of CH34. However, the strongest upregulation under this inducing conditions observed (Reith et al., 2009) was that of a genomic region (Rmet_4685 - Rmet_4682) renamed “gig” for “gold-induced genes” (Wiesemann et al., 2013). Upstream in the opposite direction are genes for the extracytoplasmic function (ECF) sigma factor RpoQ (Grosse et al., 2007), followed by the gene for a putative anti-sigma factor (Rmet_4686/7). Surprisingly, neither the *gig* cluster nor the sigma factor RpoQ are present in strain BS1 due to the lack of CMGI-D, although this strain is an isolate from a copper-gold mine and strain CH34 was isolated from a Belgian zinc decantation tank in 1976 (Mergeay et al., 1978, 1985). *C. metallidurans* was able to biomineralizes Au nanoparticles (Au at valence level of zero) by reductive precipitation from Au(I/III) -complexes and provided evidence for the bacterial contribution to the authigenic formation of secondary bacterioform gold grains and nuggets (Reith et al., 2007, 2009). The genes are coding for the periplasmic copper oxidases CopA/CopA_1_, which functions as an oxygen consuming Au(I)-oxidase that detoxifies gold complexes by a reduction pathway of Au(III)-complexes via Au(I)-intermediates to Au(0) nanoparticles in the periplasm are highly conserved in both strains (Bütof et al., 2018) (Supplementary Table S3 and Figure 11). This genome endowment in terms of copper and gold resistance factors should allow the *C. metallidurans* strain BS1, just like strain CH34, to survive in high copper and gold contaminated environments.

### The Czc – The Main Resistance Determinants for Zinc, Cobalt, and Cadmium

The Czc determinant mediating resistance to Co, Zn, and Cd is encoded on pMOL30 in *C. metallidurans* CH34 (Getaneh and Alemayehu, 2006; Rea et al., 2016) starting from MgtC until CzcP from (Rmet_5985 - Rmet_5970). Duplication of this *czcNICBADRSJE_czcP* cluster in BS1 was shown to have led to one copy being present on chromid and pBS1, respectively, and generated by mobile elements as shown in Figure 7. Some of the *czc* determinant encoded proteins, such as CzcB, CzcD, CzcR, CzcS, CzcE, CzcJ, and CzcF displayed up to 100% similarity between *C. metallidurans* BS1 and CH34 while *czc*_1_ cluster on chromid was up to 100% similar (Supplementary Table S4). CzcA, and CzcC, CzcP were also found to have up to 99% AA sequence identity in both strains. Above mentioned components were located on different loci, as BS1 harbors them on chromid and plasmid while in CH34 they were only present on pMOL30 (Supplementary Table S6 and Figure 6). The duplicate on plasmid is less similar to *czc* on pMOL30 present on type strain *C. metallidurans* CH34, as compared to *czc* on chromid in *C. metallidurans* BS1 (Supplementary Table S4). Sequence of the gene on the megaplasmid pMOL30 of CH34 encoding CzcP was only 76% similar to CzcP encoded on the plasmid (*czcP*_1_) in BS1 which has a nonsense mutation in the middle of the reading-frame.

These observations suggested that the *czc* cluster present on chromid of BS1 was more preserved and similar to the *czc* cluster on pMOL30 in CH34 than *czc* present on the pBS1 in BS1. This phenomenon of a *czc* determinant duplication was observed in *C. metallidurans* BS1 while previously a similar phenomenon was also reported in *C. metallidurans* NA4 where a large part of the resistance systems was found not to be plasmid-encoded. *C. metallidurans* NA4 was shown to have similar genome architecture in that the main large metal resistance clusters on pMOL30 were syntenic with gene clusters on NA4’s chromid instead of NA4’s plasmids (pNA4_A-D) as was also the case in BS1 (Ali et al., 2019) (Supplementary Figure S2). The *zntA*_*czcI_2_B_2_A_2__*S*_2_R_2_* core-determinant was present (DDF84_RS21985 - DDF84_RS22020) comprising structural and regulatory genes as well as encoding the P_IB__2_-type ATPase, ZntA (Legatzki et al., 2003; Scherer and Nies, 2009). The *czcICBA* cluster was referred here as a core determinant, as it is highly conserved in the genus *Cupriavidus* (Nies, 2016). These determinants have their corresponding components on the genome of CH34 with similarity of up to 99% and both determinants were present on chromid in respective strains. A recombination event (between to copies of Tn6050) rearranged the gene regions from Rmet_4598 – Rmet4687 and insert CMGI-B and D in strain CH34 led to a sequence break from locus *czcB′′* at Rmet_4469 and starting again with locus *czcB*′_*czcC* (Rmet_4596) with same pattern onward (*czcI* Rmet_4595, P_IB__2_-type ATPase Rmet_4594; ZntA) as the determinant was present in strain BS1 with 98–99% similarity index (Figure 7 and Supplementary Table S4). CMGI-B and the largest part of CMGI-D (CMGI-D′) are absent in BS1 and left the *zntA* – *czcI_2_C_2_B_2_A_2_* intact. An interesting phenomenon of genome plasticity was pointed out in *C. metallidurans* BS1, as another or forth *czc*_3_ cluster could be found on the genome of BS1 with a gene encoding a Fe-CDF member (CdfY) in same direction and close to the *mer*_2_ cluster on the chromid (Figures 7, 9). CzcA_3_ is closely related to CzcA, CzcA_1_ as well as CzcA_2_ and shared 92.8, 92.9, and 79.2% similarity on AA level. All CzcAs clustered in one branch of the phylogenetic tree in comparison to other HME-RND proteins and the *czc*_3_ cluster showed another modifying duplication during evolution (Figure 7).

### The Cnr, Chr, and Ncc – The Main Resistance Determinants for Cobalt, Nickel, and Chromium

The cobalt and nickel resistance (*cnr*) operon in CH34 was similar to that found in BS1. Both the probable structural (*cnrCBAT*) and regulatory (*cnrYXH*) genes were present on chromid but with only 93, 89, 89, 93, 85, 83, and 61% AA sequence similarity to CnrA, CnrB, CnrC, CnrT, CnrH, CnrX, and CnrY from *C. metallidurans* CH34, respectively, indicating that this determinant might have undergone an accelerated mutation rate during evolutionary adaptation (Supplementary Table S5). From the complete genome sequence of BS1, we observed that nearly all those heavy metal resistance determinants harbored on pMOL30 and pMOL28 (Monchy et al., 2007) were present on the chromid in *C. metallidurans* BS1 displaying nearly the same pattern. Four operons, *czc, cnr*, *chr*, and *mer* on the chromid in *C. metallidurans* BS1 were present side by side with one mobile genetic element (MGEs) between *czc* and *cnr*, while in type strain CH34, *czc* is present on pMOL30 and *cnr* and *chr* are present on pMOL28 (Nies, 1995; Grass et al., 2000; Monchy et al., 2007) from region DDF84_RS23090 – DDF84_RS23305 (Figure 6). The similarity of these metal determinants between *C. metallidurans* BS1 and *C. metallidurans* CH34 was up to 100% except *cnr* but with same pattern (synteny) and location upstream of the *chr* cluster as it is in strain CH34 (Supplementary Table S5). The chromid also contained *cop, sil* and *ncc* determinants from 2,055,539 bp to 2,099,418 bp on the *C. metallidurans* BS1 genome surrounded by MGEs (Figure 6). The components of the *ncc* resistance determinant such as NreB, NccC, NccB, and NccA (Nickel-Cobalt-Cadmium Resistance Protein), showed a 100% identical AA sequence in both strains, CH34 and BS1. A nonsense/frameshift mutation in NccB, indicated to be present in strain CH34 and NA1, NA4, H1130 (Van Houdt et al., 2018) was also observed to be present in BS1. The smaller segment of NccB is 100% similar to the NccB segment in strain CH34 both by nucleotide and amino acid BLAST analysis but the larger locus was 99% similar, with one arginine replaced with isoleucine in BS1. The nickel resistance protein sequence, NreB, in *C. metallidurans* CH34 (Rmet_6144) on pMOL30 displayed 100% sequence identity to the protein found on the chromid of *C. metallidurans* BS1, which also encoded *cnr* putative structural genes, *cnrCBA*. The presence of the *czc* and *cnr* operons encoded on the same locus supported the idea of an ancestral cobalt or cobalt-zinc resistance operon that evolved divergently by duplication and acquired additional specificities to become *cnr* and *czc* (Janssen et al., 2010). Similar to *C. metallidurans* CH34, the chromate resistance (*chr*) determinant was located upstream of the *cnr* operon in *C. metallidurans* BS1. Using the RAST functional based comparison, it was determined that both *C. metallidurans* BS1 and CH34 contained genes encoding all the proteins (ChrABCEF) responsible for resistance to chromium compounds. A Blast search of the ChrI sequence of *C. metallidurans* CH34 obtained from NCBI against the genome of *C. metallidurans* BS1 in RAST showed that ChrI in *C. metallidurans* BS1 had only 44% AA sequence similarity to ChrI from *C. metallidurans* CH34.

In addition, the *nimBAC* locus encoding an RND-system was suggested to putatively play a role in resistance to Ni^2+^ and Co^2+^. The similarity index of *nimBAC* is 100%-96% as indicated in Supplementary Table S6. The *nimBAC* operon is suspected to be inactivated by a transposon insertion in CH34 but it is intact in NA1, NA4 and H1130 (Van Houdt et al., 2018).

### Mercury and Arsenic Resistance Determinants

Three mercury (Hg^2+^) resistance operon are encoded on the genome of BS1 and four in CH34, including six genes coding for MerR < – > MerTPC/ADE (Supplementary Figure S3 and Supplementary Table S6). Eleven genes encoding proteins associated with arsenic (AsO_4_^3–^/AsO_3_^3–^) resistance including ArsHR, 2 ACR3 (arsenical resistance protein), and 5 ArsC while 13 proteins were found associated with chromium (CrO_4_^2–^) resistance including ChrABCEF (Supplementary Table S6 and Figure 6) could be detected on the genome of *C. metallidurans* BS1. In the genome of *C. metallidurans* BS1 there was a comparable number of MerR encoding genes with 97–100% similarity at the amino acid level to CH34 (Supplementary Figure S3). These included CupR (activator of CupA) and PbrR (activator of PbrA) and a not further characterized MerR regulator (locus tag: DDF84_RS1704_BS1/Rmet_3456_CH34) which clustered in the phylogenetic tree with the zinc-, cadmium- and lead-dependent homologs PbrR, CadR from *C. metallidurans* CH34 and CadR from *Paraburkholderia xenovorans* LB400 as well as ZntR from *E. coli* (Brocklehurst et al., 1999; Taghavi et al., 2009; Van Houdt et al., 2009). The three encoded homologous MerR regulators (locus tag: DDF84_RS08325, DDF84_RS22830 and DDF84_RS23300) of the mercury resistance clusters (MerR < - > MerTPC/ADE) are transposon duplicates with 100% similarity on the amino acid level including MerR2 (locus tag: Rmet_6171) of CH34. Two further *mer* clusters including the *mer*R_3_ and *mer*R_4_ genes from CH34 were missing in strain BS1 due to the absence of CMGI-1 and a transposon insertion on pMOL30 (Table 4). The Mer-type regulator SoxR, involved in the oxidative stress response, was present in both strains with a similarity of 98% in both strains, as well as homologs of as yet uncharacterized members of this protein family (Supplementary Figure S3).

### Megaplasmid pBS1 in BS1

Plasmids in *C. metallidurans* CH34 are of prime importance as most metal resistance determinants are encoded on pMOL30 and pMOL28 (Nies, 1999) but this is not the case in *C. metallidurans* BS1. The plasmid in BS1 only contains one duplicate copy of *czcNICBADRSEJ_czcP* cluster and abundant hypothetical proteins and mobile genetic elements. Genes encoding putative transfer functions TraW^∗^, TrU^∗^, TrbC^∗^, (^∗^orthologs of IncF plasmids) TraD, and TraN are predicted to be involved in plasmid transfer, were present on plasmid –pBS1 DNA displaying the same pattern as on pMOL30 in CH34. The pBS1 plasmid displayed more similarities with pMOL30 by having similar plasmid maintenance systems while, the pBS1 plasmid showed no similarities with pMOL28 with the sole exception of a 3.5 kbp region [mobile element; Rmet_6337 (DDF84_031600) – Rmet_6334 (DDF84_031615)]. Three copies of Polymyxin resistance protein amino-arabinose transferase ArnT were found on the chromid in BS1 and one copy on plasmid out of four ArnT locus in total while it is a component of pMOL30 in CH34. This last-resort antibiotic is affective against Gram negative bacteria by permeabilizing its outer membrane using electrostatic interactions between amino group of polymyxins and negatively charged glucosamine and 3-deoxy-D-manno-oct-2-ulosonic acid (Kdo) sugars of lipid A (Tseng et al., 1999). We believe that ArnT present in BS1 might be involved in outer membrane modifications in response to heavy metal stress. Genome plasticity might be responsible for relocating many heavy metal resistance determinants to the chromid. It could be suspected these determinants were fixed on the chromid as strain BS1 might be inhabiting an environment with high levels of heavy metals for a long time. It is predicted that transformation frequency and conjugation frequency is influenced and possibly induced by metals to select for increased survival in the environment. Mobile genetic elements (MGEs) play a vital role in evolutionary processes and are responsible for transferring genetic information among and within bacterial species. A significant role of MGEs is to harbor putative virulence factors and genes encoding molecules and functions that confer resistance to heavy metals and antibiotics while providing opportunities for Horizontal Gene Transfer.

## Conclusion

This study presents the complete genome sequence and annotation of *C. metallidurans* BS1, a highly heavy metal resistant bacterium. The 7,164,523 bp complete genome sequences of *C. metallidurans* BS1 contains three replicons present as one chromosome, one chromid and one plasmid (pBS1). Putative heavy metal resistance determinants of *C. metallidurans* BS1 were largely similar to those present on the genome of *C. metallidurans* CH34. A noticeable difference was observed in *C. metallidurans* BS1 as major determinants of metal detoxification systems were located on the chromid instead of the plasmids (pMOL30 and pMOL28) in *C. metallidurans* CH34. Known gold–copper detoxification systems (CupR/CueR, CupA, CupC and CopA) found in *C. metallidurans* CH34 were also present in *C. metallidurans* BS1. Two prophages detected on the genome are probably intact and one very likely intact. Metal resistance determinants from different origins in BS1 displayed critical differences between *C. metallidurans* BS1 and CH34. Future studies would be aimed to explore the mechanisms involved in the interplay between gold and copper in gold–copper detoxification systems. In addition, one future aim will be to better understand the evolutionary adaptation to the auriferous environment of isolation on the genetic level and further reconstruct genomic rearrangements.

## Data Availability Statement

The data used to support the findings of this study are included within the article. Please check https://www.ncbi.nlm.nih.gov/assembly/GCA_003260185.2 for more details.

## Author Contributions

SM and SB performed the laboratory experiments, analyzed the annotated genome sequence, submitted genome to NCBI, wrote the draft manuscript, and participated in the design of the experiments. MH carried out the genomic characterization and constructed the phylogenetic trees. IF, YL, JS, and JX performed the description of the sampling environment, sampling, isolation and microbiological characterization of the bacteria. CZ, SM, and SB carried out the isolation and purification of the genomic DNA. CR, RF, SZ, and IF participated in the design of the study and revised the manuscript. All authors read and approved the final manuscript.

## Conflict of Interest

The authors declare that the research was conducted in the absence of any commercial or financial relationships that could be construed as a potential conflict of interest.

## Footnotes

1 http://www.illumina.com.cn/

2 https://github.com/PacificBiosciences/pbdagcon

3 https://www.broadinstitute.org/gatk/

4 http://www.ebi.ac.uk/genomes/plasmid.html

5 www.ncbi.nlm.nih.gov

6 https://www.geneious.com

7 http://rfam.sanger.ac.uk/

8 http://phast.wishartlab.com/
