# Supplementary material for: Comparative Insights Into the Complete Genome Sequence of Highly Metal Resistant Cupriavidus metallidurans Strain BS1 Isolated From a Gold–Copper Mine
Source: Front Microbiol. 2020 Feb 7;11:47. doi: 10.3389/fmicb.2020.00047 (PMC7019866; doi:10.3389/fmicb.2020.00047)
Supplement: Supplementary file 1 [file Data_Sheet_1.PDF]

Supplementary Data

Table S1: Insertion sequence elements distribution in *Cupriavidus metallidurans* BS1

**Elements**	**Family**	**Size**	**similarity**	**CHR1**	**CHR2**	**pBS1**	**origin**
ISRme10	**IS30**	3031	100%		1		*Cupriavidus metallidurans*
ISRme13	**IS3**	2537	99%	1	1	1	*Cupriavidus metallidurans*
IS1071	**Tn3**	6308	99%		1		*Alcaligenes* sp.
ISRme9	**IS21**	4260	95%	1	1		*Cupriavidus metallidurans*
ISPa38	**Tn3**	4218	93%	1	1		*Pseudomonas aeruginosa*
TnAs3	**Tn3**	4094	90%	1	1		*Aeromonas salmonicida*
TnShfr1	**Tn3**	2799	90%	1	1		*Shewanella frigidimarina*
ISShes11	**Tn3**	3170	90%	1	1		*Shewanella* spp.

[1] Percentage nucleotide sequence similarity with insertion sequence (IS) elements found by using ISFinder (Siguier, 2006)

Table S2: AA (amino acid) similarity index of the sigma factors between *Cupriavidus metallidurans* BS1 and *Cupriavidus metallidurans* CH34

number	name	replicon	CH34<br>Rmet_no. (NCBI)	BS1<br>Locus_tag (NCBI)	Similarity (%) [a]
**ICF1**	RpoD	chromosome	Rmet_2606	DDF84_RS13695	99.6
**ICF2**	RpoN	chromosome	Rmet_0303	DDF84_RS01545	99.2
**ICF3**	RpoS	chromosome	Rmet_2115	DDF84_RS11235	99.5
**ICF4**	$RpoD_2$	chromid	Rmet_4661	-	-
**ICF5**	RpoH	chromosome	Rmet_0272	DDF84_RS01390	100
**ICF6**	RpoF_FliA	chromid	Rmet_3702	DDF84_RS28670	100
**ECF1**	RpoJ	chromid	Rmet_4499	DDF84_RS22465	99
**ECF2**	RpoK	chromid	Rmet_4001	DDF84_RS19655	97.6
**ECF3**	RpoI	chromosome	Rmet_1120	DDF84_RS05700	100
**ECF4**	RpoL	chromosome	Rmet_3280	DDF84_RS16615	99.4
**ECF5**	RpoM	chromid	Rmet_5400	DDF84_RS27330	99.4
**ECF6**	RpoO	chromosome	Rmet_0597	DDF84_RS02995	98.1
**ECF7**	RpoP	chromosome	Rmet_1648	DDF84_RS07630	98.0
**ECF8**	RpoE	chromosome	Rmet_2425	DDF84_RS12830	100
**ECF9**	CnrH	$pMOL28_{CH34}$ / $chromid_{BS1}$	Rmet_6207	DDF84_RS23175	85.9
**ECF10**	RpoQ	chromid	Rmet_4686	-	-
**ECF11**	RpoR	chromosome	Rmet_0910	DDF84_RS04560	99.5
**ECF12**	SigJ	chromid	Rmet_3844	DDF84_RS18870	98.3
**ECF13**	SigX	chromid	-	DDF84_RS21190	-

a) Similarity percentage of the amino acid sequence of the sigma factors between chromosome & chromid (CHR2) of BS1 and chromosome, chromid or pMOL28 of CH34; ICF (sigma factors of the intracytoplasmic function), ECF (sigma factors of the extracytoplasmic function)

Table S3*: AA (Amino Acid) similarity index of Cop determinants between *Cupriavidus metallidurans* BS1 and *Cupriavidus metallidurans* CH34*

**Resistant Determinant**	**pMOL30(CH34)** **Locus_tag (NCBI)**	**chromid(BS1)** **Locus_tag (NCBI)**	**Similarity (%)** [a] AA sequence
**CopV**	Rmet_6105	DDF84_RS27645	100
**CopT**	Rmet_6106	DDF84_RS27650	100
**CopM**	Rmet_6107	DDF84_RS27660	100
**CopK**	Rmet_6108	DDF84_RS27665	100
**CopN**	Rmet_6109	DDF84_RS27670	100
**CopS**	Rmet_6110	DDF84_RS27675	100
**CopR**	Rmet_6111	DDF84_RS27680	100
**CopA**	Rmet_6112	DDF84_RS27685	99.8
**CopB**	Rmet_6113	DDF84_RS27690	78.6
**CopC**	Rmet_6114	DDF84_RS27695	100
**CopD**	Rmet_6115	DDF84_RS27700	100
**CopI**	Rmet_6116	DDF84_RS27705	100
**CopJ**	Rmet_6117	DDF84_RS27710	100
**CopG**	Rmet_6118	DDF84_RS27715	100
**CopF**	Rmet_6119	DDF84_RS27720	100
**CopL**	Rmet_6120	DDF84_RS27730	100
**CopQ**	Rmet_6121	DDF84_RS27735	100
**CopH**	Rmet_6122	DDF84_RS27740	100
**CopE**	Rmet_6123	DDF84_RS27745	100
**Resistant Determinant**	**chromid(CH34)** **Rmet_no. (NCBI)**	**chromid(BS1)** **Locus_tag (NCBI)**	**Similarity (%)** [a]
**$CopS_1$**	Rmet_5673	DDF84_RS30595	99
**$CopR_1$**	Rmet_5672	DDF84_RS30600	100
**$CopA_1$**	Rmet_5671	DDF84_RS30605	95.4
**$CopB_1$**	Rmet_5670	DDF84_RS30610	93
**$CopC_1$**	Rmet_5669	DDF84_RS30615	97.7
**$CopD_1$**	Rmet_5668	DDF84_RS30620	97.3
**$CopA_x$**	Rmet_4701	DDF84_RS23375	98.3
**$CopA_y$**	Rmet_4702	DDF84_RS23380	99.6

a) Similarity percentage of the amino acid sequence of the Cop resistance proteins between Chromid (CHR2) of BS1 and chromid or pMOL30 of CH34

Table S4*: Similarity index of Czc determinants between *Cupriavidus metallidurans* BS1 and *Cupriavidus metallidurans* CH34*

**Resistant Determinant**	***czc_1* (BS1)/ *czc* (CH34)**		***czc* (BS1)/ *czc* (CH34)**		***czc_1* (BS1)/ *czc* (BS1)**	
**replicon**	**chromid(BS1) / pMol30$_{(CH34)}$ (NCBI locus tag)**	**Similarity (%)[a]**	**Plasmid(BS1) / pMol30$_{(CH34)}$ (NCBI locus tag)**	**Similarity (%)[b]**	**chromid(BS1) / Plasmid(BS1) (NCBI locus tag)**	**Similarity (%) [c]**
**CzcP**	DDF84_023065/ Rmet_5970	100	DDF84_RS31480/ Rmet_5970	76	DDF84_023065/ DDF84_RS31480	76
**FlgB**	DDF84_023070/ Rmet_5971	100	DDF84_RS31485/ Rmet_5971	96.4	DDF84_023070/ DDF84_RS31485	96.4
**OMP**	DDF84_023075/ Rmet_5974	99	DDF84_RS31490/ Rmet_5974	93	DDF84_023075/ DDF84_RS31490	99
**CzcJ**	DDF84_023080/ Rmet_5975	100	DDF84_RS31495/ Rmet_5975	96	DDF84_023080/ DDF84_RS31495	96
**CzcE**	DDF84_023085/ Rmet_5976	100	DDF84_RS31500/ Rmet_5976	73	DDF84_023085/ DDF84_RS31500	72
**CzcS**	DDF84_023090/ Rmet_5977	100	DDF84_RS31505/ Rmet_5977	85	DDF84_023090/6 DDF84_RS31505	85
**CzcR**	DDF84_023095/ Rmet_5978	99	DDF84_RS31510/ Rmet_5978	96	DDF84_023095/ DDF84_RS31510	97
**CzcD**	DDF84_023100/ Rmet_5979	100	DDF84_RS31515/ Rmet_5979	91	DDF84_023100/ DDF84_RS31515	91
**CzcA**	DDF84_023105/ Rmet_5980	99	DDF84_RS31520/ Rmet_5980	97	DDF84_023105/ DDF84_RS31520	97
**CzcB**	DDF84_023100/ Rmet_5981	100	DDF84_RS31525/ Rmet_5981	93	DDF84_023110/ DDF84_RS31525	93
**CzcC**	DDF84_023115/ Rmet_5982	99	DDF84_RS31530/ Rmet_5982	95	DDF84_023115/ DDF84_RS31530	95
**CzcI**	DDF84_023120/ Rmet_5983	100	N.A	N.A	N.A	N.A
**C.H.P**	DDF84_023125/ Rmet_6368	100	N.A	N.A	N.A	N.A
**CzcN**	DDF84_023130/ Rmet_5984	99	DDF84_RS31535/ Rmet_5984	95	DDF84_023130/ DDF84_RS31535	95
**MgtC**	DDF84_023135/ Rmet_5985	99	DDF84_RS31550/ Rmet_5985	93	DDF84_023135/ DDF84_RS31550	92
**hyp**	DDF84_023140/ Rmet_5986	100	N.A	N.A	N.A	N.A

a) Similarity percentage of Czc between Chromosome 2 of BS1 and pMOL30 of CH34: **b)** Similarity percentage of Czc between of plasmid of BS1 and pMOL30 of CH34: **c)** Similarity percentage of Czc between Chromosome 2 of BS1 and plasmid of BS1

Table S5*: AA (Amino Acid) similarity index of Cnr and Chr$_1$ determinants between *Cupriavidus metallidurans* BS1 *and Cupriavidus metallidurans* CH34

**Resistant Determinant**	**pMOL28$_{(CH34)}$** **Rmet_no. (NCBI)**	**chromid$_{(BS1)}$** **Locus_tag (NCBI)**	**Similarity (%)** [a]
**Hyp** **(hypothetical protein)**	Rmet_6198	DDF84_RS23215	None
**ChrX$_1$**	Rmet_6199	DDF84_RS23210	91
**ChrE$_1$**	Rmet_6200	DDF84_RS23205	96
**SodC**	Rmet_6201	DDF84_RS23200	99
**ChrA$_1$**	Rmet_6202	DDF84_RS23195	100
**ChrB$_1$**	Rmet_6203	DDF84_RS23190	100
**ChrI$_1$**	Rmet_6204	- (absent)	
**CnrY**	Rmet_6205	DDF84_RS23185	61
**CnrX**	Rmet_6206	DDF84_RS23180	83
**CnrH**	Rmet_6207	DDF84_RS23175	85
**CnrC**	Rmet_6208	DDF84_RS23170	89
**CnrB**	Rmet_6209	DDF84_RS23165	89
**CnrA**	Rmet_6210	DDF84_RS23160	93
**CnrT**	Rmet_6211	DDF84_RS23155	93

a) Similarity percentage of Cnr and Cop between chromid (CHR2) of BS1 and pMOL28 of CH34

Table S6: Comparison of metal determinants between *C. metallidurans* BS1 and type strain *C. metallidurans* CH34 through multiple export systems in Heavy Metals Resistance gene clusters

**Type**[a]	Gene cluster[b]	Replicon CH34	Replicon BS1	Rmet_no. (NCBI) (CH34)	Locus_tag (NCBI) (BS1)	Similarity (%) by Nt [c]	Similarity (%) by A.A [d]	Metals	Mechanism[e]	Function[f]	Ref.
PPR	***cdfX***<br>*cadR_cadA_*<br>*cadC*<br>*merR*(§)	CHR1	-	Rmet_2299,<br>Rmet_2302–<br>2304,<br>Rmet_3456	N.A<br>N.A<br><br>DDF84_017405	N.A<br>N.A<br><br>99%	N.A<br>N.A<br><br>100%	Zn^{2+}<br>Cd^{2+}	ExP, pCDF<br>ExP, P-type	+	(Van Houdt et al., 2009)
PPR	***pbrUb***|*UaTR*<br>*pbrABCD*(#)	pMOL30	Chromid	Rmet_6180<br>Rmet_5944<br>Rmet_5945<br>Rmet_5946<br>Rmet_5947<br>Rmet_5948<br>Rmet_5949	DDF84_RS22880<br>DDF84_RS22880<br>DDF84_RS22885<br>DDF84_RA22890<br>DDF84_RA22895<br>DDF84_RS22900<br>DDF84_RS22905	100%<br>100%<br>100%<br>100%<br>100%<br>100%<br>100%	100%<br>N.A<br>100%<br>100%<br>100%<br>100%<br>100%	Pb^{2+}	ExP, seq	+	(Borremans et al., 2001)
	cupRAC	chromosome	chromosome	Rmet_3523<br>Rmet_3524<br>Rmet_3525	DDF84_ RS 17860<br>DDF84_ RS 17865<br>DDF84_ RS 17870	97%<br>98%<br>98%	99%<br>98%<br>100%	Ag^{+},<br>Cu^{+},<br>Cu^{2+}	ExP, P-type	+	(Monchy et al., 2006; Wiesemann et al., 2013)
	merRTPA'A"	CHR1	-	Rmet_2312-<br>2315	N.A	N.A	N.A	Hg^{2+}	HgRed	?	(Van Houdt et al., 2009)
PPR	*merRTPADE urf-2*	pMOL28	Chromid & chromosome	Rmet_6344<br>Rmet_6345<br>Rmet_6346<br>Rmet_6183<br>Rmet_6184<br><br>Rmet_6185<br><br>Rmet_6186	DDF84_RS22830 /<br>RS08325<br>DDF84_RS22835 /<br>RS08320<br>DDF84_RS22840 /<br>RS08315<br>N.A<br>DDF84_RS22850 /<br>RS08305<br>DDF84_RS22855 /<br>RS08300<br>DDF84_RS22860 /<br>RS08295	100%,100%<br>100%,100%<br>100%,100%<br>N.A<br>100%,100%<br><br>100%,100%<br><br>100%,100%	100%,100%<br>100%,100%<br>100%,100%<br>N.A<br>100%,100%<br><br>100%,100%<br><br>100%,100%	Hg^{2+}	HgRed	+	(Diels et al., 1985)
PPR	*merRTPADE urf-2*	pMOL30	Chromid & chromosome	Rmet_6171<br>Rmet_6172	DDF84_RS22830 /<br>RS08325	100%,100%<br>100%,100%	100%,100%<br>100%,100%	Hg^{2+}	HgRed	+	(Diels et al., 1985)

				Rmet_6173<br>Rmet_6174<br>Rmet_6175<br>Rmet_6176<br>Rmet_6177	DDF84_RS22835 / RS08320<br>DDF84_RS22840 / RS08315<br>DDF84_RS22845 / RS08310<br>DDF84_RS22850 / RS08305<br>DDF84_RS22855 / RS08300<br>DDF84_RS22860 / RS08295	100%,100%<br>100%,100%<br>100%,100%<br>100%,100%<br>100%,100%	100%,100%<br>100%,100%<br>100%,100%<br>100%,100%<br>100%,100%				
PPR	*merRTΔP*	pMOL30	CHR2	Rmet_5990–5992	N.A	N.A	N.A		HgTra	?	(Monchy et al., 2007)
**PPR**	*chrBAF*	Plasmid	chromid	Rmet_3866<br>Rmet_3865<br>Rmet_3864	DDF84_ RS 18975<br>DDF84_ RS 18970<br>DDF84_ RS 18965	98%<br>98%<br>97%	99%<br>99%<br>97%	$CrO4^{2-}$	ExChr	?	(Juhnke et al., 2002, 2004)
PPR	*chrIBACEF**ONPYZ***	pMOL28	N.A	Rmet_6204-6194	N.A	N.A	N.A		ExChr,FS,MFS	+	(Nies et al., 1990)
NPR	*ars**P**HC₁BC₂I<u>R</u>**M***	chromosome	chromosome	Rmet_0327<br>Rmet_0328<br>Rmet_0329<br>Rmet_0330<br>Rmet_0331<br>Rmet_0332<br>Rmet_0333<br>Rmet_0334	DDF84_ RS 01615<br>DDF84_ RS 01620<br>DDF84_ RS 01625<br>DDF84_ RS 01630<br>DDF84_ RS 01635<br>DDF84_ RS 01640<br>DDF84_ RS 01645<br>DDF84_ RS 01650	96%<br>97%<br>98%<br>96%<br>98%<br>96%<br>96%<br>98%	95%<br>95%<br>98%<br>97%<br>98%<br>98%<br>94%<br>98%	$HAsO4^{2-}$<br>AsO^{2-}	AsRed (ExAs)	+	(Zhang et al., 2009)
PPR	*copSRABCD*	Plasmid	chromid	Rmet_5673<br>Rmet_5672<br>Rmet_5671<br>Rmet_5670<br>Rmet_5669<br>Rmet_5668	DDF84_ RS 30595<br>DDF84_ RS 30600<br>DDF84_ RS 30605<br>DDF84_ RS 30610<br>DDF84_ RS 30615<br>DDF84_ RS 30620	98%<br>97%<br>97%<br>94%<br>98%<br>97%	98%<br>100%<br>95%<br>93%<br>97%<br>97%	Cu^{2+}	ExCop / seq	+	(Wiesemann et al., 2013)
PPR	*cop**V**TK**MN**SRABC<br>DI**J**GF**O**L**Q**H**EW***	pMOL30	chromid	Rmet_6105<br>Rmet_6106<br>Rmet_6107<br>Rmet_6108<br>Rmet_6109<br>Rmet_6110<br>Rmet_6111<br>Rmet_6112	DDF84_ RS27645<br>DDF84_ RS27650<br>DDF84_ RS27660<br>DDF84_ RS27665<br>DDF84_ RS27670<br>DDF84_ RS27675<br>DDF84_ RS27680<br>DDF84_ RS27685	100%<br>100%<br>100%<br>100%<br>100%<br>100%<br>100%<br>99%	100%<br>100%<br>100%<br>100%<br>100%<br>100%<br>100%<br>99%	Cu^{2+}	ExCop / ExP / seq<br>+		(Monchy et al., 2006)

				Rmet_6113	DDF84_ RS27690	99%	100%				
				Rmet_6114	DDF84_ RS27695	100%	100%				
				Rmet_6115	DDF84_ RS27700	100%	100%				
				Rmet_6116	DDF84_ RS27705	100%	100%				
				Rmet_6117	DDF84_ RS27710	100%	100%				
				Rmet_6118	DDF84_ RS27715	100%	100%				
				Rmet_6119	DDF84_ RS27720	100%	100%				
				Rmet_6382	DDF84_ RS27725	100%	100%				
				Rmet_6120	DDF84_ RS27730	100%	100%				
				Rmet_6121	DDF84_ RS27735	100%	100%				
				Rmet_6122	DDF84_ RS27740	100%	100%				
				Rmet_6123	DDF84_ RS27745	100%	100%				
				Rmet_6124	DDF84_ RS27750	100%	100%				
	zntA	chromid	chromid	Rmet_4594	DDF84_ RS 22020	97%	98%	Cd2+,	ExHME1, ExP	(+)	(Nies et al.,
	czcICΔBA ubiG czc			Rmet_4595	DDF84_ RS 22015	98%	98%	Zn2+,			2006)
	*SR**L**| **hns mmmQ***			Rmet_4596	DDF84_ RS 22010	98%	99%	Co2+			
				Rmet_4597	DDF84_ RS 22005	95%	N.A				
				Rmet_4469	DDF84_ RS 22005	98%	N.A				
				Rmet_4468	DDF84_ RS 22000	97%	98%				
				Rmet_4467	DDF84_ RS 21995	98%	100%				
				Rmet_4466	DDF84_ RS 21990	97%	99%				
				Rmet_4465	DDF84_ RS 21985	99%	100%				
				Rmet_4464	DDF84_ RS 21980	95%	95%				
				Rmet_4463	N.A	N.A	N.A				
				Rmet_4462	DDF84_ RS 21975	99%	98%				
				Rmet_4461	DDF84_ RS 21970	96%	98%				
	mgtCczcNICBADR	pMOL30	chromid+	Rmet_5985	DDF84_ RS 23135	98% / 86%	99% / 92%		ExHME1 /	+	(Nies and
	*SE**J ompP** czcP*		Plasmid	Rmet_5984	DDF84_ RS 23130	97% / 83%	98%,95%		ExP		Silver, 1989;
				Rmet_5983	DDF84_ RS 23120	100% / 88%	N.A / N.A				Grosse et al.,
				Rmet_5982	DDF84_ RS 23115	99% / 87%	99% / 94%				1999, 2004)
				Rmet_5981	DDF84_ RS 23110	100% / 83%	100%,93%				
				Rmet_5980	DDF84_ RS 23105	99% / 87%	99% / 96%				
				Rmet_5979	DDF84_ RS 23100	100% / 87%	100% / 91%				
				Rmet_5978	DDF84_ RS 23095	100% / 87%	99% / 96%				
				Rmet_5977	DDF84_ RS 23090	100% / 81%	100%,84%				
				Rmet_5976	DDF84_ RS 23085	100% / 87%	100% / 71%				
				Rmet_5975	DDF84_ RS 23080	100% / 89%	100% / 95%				
				Rmet_5974	DDF84_ RS 23075	99% / 84%	99% / 92%				
				Rmet_5970	DDF84_ RS 23065	99% / 82%					

	cnrYXHCBAT	pMOL28	chromid	Rmet_6205<br>Rmet_6206<br>Rmet_6207<br>Rmet_6208<br>Rmet_6209<br>Rmet_6210<br>Rmet_6211	N.A<br>N.A<br>N.A<br>DDF84_ RS 23170<br>DDF84_ RS 23165<br>DDF84_ RS 23160<br>DDF84_ RS 23155	N.A<br>N.A<br>N.A<br>82%<br>80%<br>83%<br>87%	N.A<br>N.A<br>N.A<br>86%<br>85%<br>93%<br>93%		ExHME2 / CDF	+	(Liesegang et al., 1993; Grass et al., 2000)
	nccCB"B'A nreB ***mmrQ***	pMOL30	chromid	Rmet_6148<br>Rmet_6147<br>Rmet_6146<br>Rmet_6145<br>Rmet_6148<br>Rmet_6143	DDF84_ RS 27860<br>DDF84_ RS 27855<br>DDF84_ RS 27855<br>DDF84_ RS 27850<br>DDF84_ RS 27845<br>DDF84_ RS 27840	100%<br>100%<br>100%<br>100%<br>100%<br>100%	100%<br>100%<br>100%<br>100%<br>100%<br>100%		ExHME2 / MFS	(+)	(Nies et al., 2006)
	nimBA|AC (*)	pMOL30	chromid+ CHR1	Rmet_5682<br>Rmet_5681<br>Rmet_5680<br>Rmet_5679<br>Rmet_5678<br>Rmet_5677	DDF84_ RS 30565<br>DDF84_ RS 30570<br>DDF84_RS18360 / RS09905<br>DDF84_RS18360 / RS09900<br>DDF84_ RS 30570<br>DDF84_ RS 30575	100%<br>98% /<br>100% / 100%<br>100% / 100%<br>98% / 81%<br>96%	99%<br>N.D.A<br>100%,100%<br>100%,100%<br>N.D.A /<br>N.A / 97%	Ni2+ / Co2+	ExHME3b / ExP	+	(Nies, 2016)
NPR	*hmzRS yodB hmzBΔA*	CHR1	N.A	Rmet_3016–3011	N.A	N.A	N.A	divalent metals	ExHME3b	-	(Van Houdt et al., 2009; Nies, 2016)
NPR	*hmvCBΔA*	chromid	chromid	Rmet_3836<br>Rmet_3837<br>Rmet_3838	DDF84_ RS 18835<br>DDF84_ RS 18840<br>DDF84_ RS 18845	97%<br>98%<br>98%	99%<br>99%<br>N.D.A		ExHME3a	-	(Nies et al., 2006)
**NPR**	*zniABCSR zne**P**RSCAB*	chromid	chromid	Rmet_5319<br>Rmet_5320<br>Rmet_5321<br>Rmet_5322<br>Rmet_5323<br>Rmet_5325<br>Rmet_5326<br>Rmet_5327<br>Rmet_5328<br>Rmet_5329<br>Rmet_5330	DDF84_ RS 26925<br>DDF84_ RS 26930<br>DDF84_ RS 26940<br>DDF84_ RS 26945<br>DDF84_ RS 26955<br>DDF84_ RS 26960<br>DDF84_ RS 26960<br>DDF84_ RS 26965<br>DDF84_ RS 26970<br>DDF84_ RS 26975<br>DDF84_ RS 26980	98%<br>97%<br>97%<br>98%<br>97%<br>97%<br>95%<br>97%<br>98%<br>97%<br>98%	99%<br>99%<br>99%<br>99%<br>99%<br>98%<br>100%<br>96%<br>97%<br>99%<br>98%		ExHME3a / MSF	?	(Nies et al., 2006; Nies, 2016; Wiesemann et al., 2017)
**NPR**	***hmyCB*| *hmy*(£)	chromid	chromid	Rmet_4120<br>Rmet_4121<br>Rmet_4122<br>Rmet_4123	DDF84_ RS 20220<br>DDF84_ RS 20225<br>N.A<br>DDF84_ RS 20230	98%<br>98%<br>81%<br>99%	99%<br>99%<br>N.A<br>99%		ExHME3b	+	(Nies, 2016)

	*cus**D**CBAF*	Plasmid	chromid	Rmet_5030<br>Rmet_5031<br>Rmet_5032<br>Rmet_5033<br>Rmet_5034	DDF84_ RS 25225<br>DDF84_ RS 25230<br>DDF84_ RS 25235<br>DDF84_ RS 25240<br>DDF84_ RS 25245	98%<br>98%<br>97%<br>97%<br>95%	98%<br>98%<br>98%<br>98%<br>95%	Ag^+,<br>Cu^+,<br>Cu^{2+}	ExHME4	+	(Mergeay et al., 2003)
	*sil**D**CBA cus**ΔF***	pMOL30	chromid	Rmet_6133<br>Rmet_6134<br>Rmet_6135<br>Rmet_6136<br>Rmet_5953	DDF84_ RS 27795<br>DDF84_ RS 27800<br>DDF84_ RS 27805<br>DDF84_ RS 27810<br>DDF84_ RS 22925	100%<br>100%<br>99%<br>100%<br>100%	100%<br>100%<br>99%<br>100%<br>100%		ExHME4	(+)	(Monchy et al., 2007; Nies, 2016)
**NPR**	*agrCBARS*	CHR1	CHR1	Rmet_1748<br>Rmet_1749<br>Rmet_1750<br>Rmet_1751<br>Rmet_1752	DDF84_ RS 08080<br>DDF84_ RS 08085<br>DDF84_ RS 08090<br>DDF84_ RS 08095<br>DDF84_ RS 08100	95%<br>95%<br>96%<br>97%<br>98%	96%<br>97%<br>98%<br>99%<br>99%		ExHAE	?	(Monchy et al., 2007)

a) chromosomal HMR resistance loci with genes that have a counterpart with a homologous function on a plasmid are called partially plasmid redundant (PPR), other loci are not- plasmid redundant (NPR).

b) Underlined genes are regulators; bold genes are new in respect to an earlier report geneparts created by a frameshift are indicated by 'and''; IS- or Tn-insertions are indicated by a pipe symbol (|); truncated genes are indicated with a delta (Δ) symbol; (§), pbrR3 acts on *zntA* (Rmet_4594); (#),insertion of Tn6049 in *pbrU*;(ï), *czcB* truncated by Tn6050-mediated rearrangement (inversion) and insertion of IS1088 between *czcL* and *hns*; (*), insertion of ISRme3 in *nimA*;(£),insertion of IS1088 between *hmyB* and *hmyA*.

c) Similarity percentage with nucleotide (Nt)

d) similarity percentage with amino acid (A.A)

e) Mechanisms of HM detoxification: ExP, efflux (P1-ATPase); pCDF, putative CDF-like; seq, periplasmic sequestration; HgRed, reduction of Hg^{2+} into Hg^0 which then volatilizes out of the cell; HgTra, tentative transport of Hg^{2+}; ExChr, transport of chromate; FS, Fe-SOD (chrC); MFS, major facilitator superfamily permease (i.e., chrP); CDF, cation diffusion facilitator; ExHME, efflux by RND-HME type 1 to 4 (see text); ExHAE, efflux by HAE-RND (unknown substrate); ExCop, extrusion of Cu^{2+};AsRed (ExAs), reduction of $HAsO_4{}^{2-}$ into AsO_2^- and efflux of AsO_2^-.

f) Functionality; +, active; (+), partly active; ?, unknown; -, inactive. doi:10.1371/journal.pone.0010433.t005

Supplementary Figures

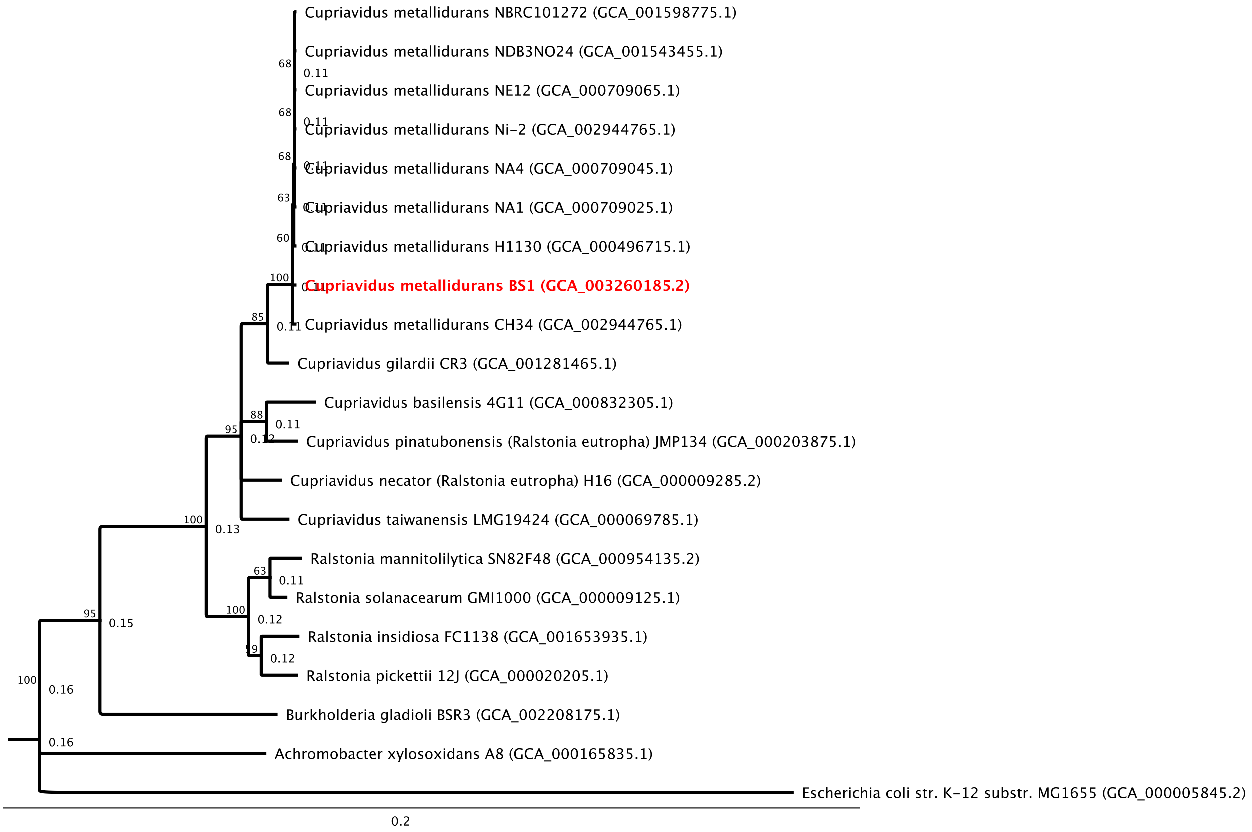

Figure_S1: Molecular Phylogenetic tree based on the 16S rRNA gene (1,533 bp) sequences highlighting the position of *C. metallidurans* strain BS1 relative to other type and non-type strains of the genus *Cupriavidus, Ralstonia, Burkholderia and Achromobacter*. GenBank assembly accession numbers are shown in parenthesis. The evolutionary history was inferred by using the Geneious prime 2020 0.4. ([Geneious] Alignment Tree Builder) ([https://www.geneious.com]), global alignment with free gaps, Identity (1.0/0.0), gap open penalty 12, gap extension penalty 3, 2 refinement iterations, Genetic Distance Model _ Jukes-Cantor, Neighbor-Joining, Resampling Method – Bootstrap; 100). A sequence from *Escherichia coli* strain K-12 substr. MG1655 was used as out group. The tree is drawn to scale, with branch lengths calculated using the average pathway method; the scale bar corresponds to the number of substitutions per site, the branch labels are consensus Support (%) and Node Heights (Kearse et al., 2012).

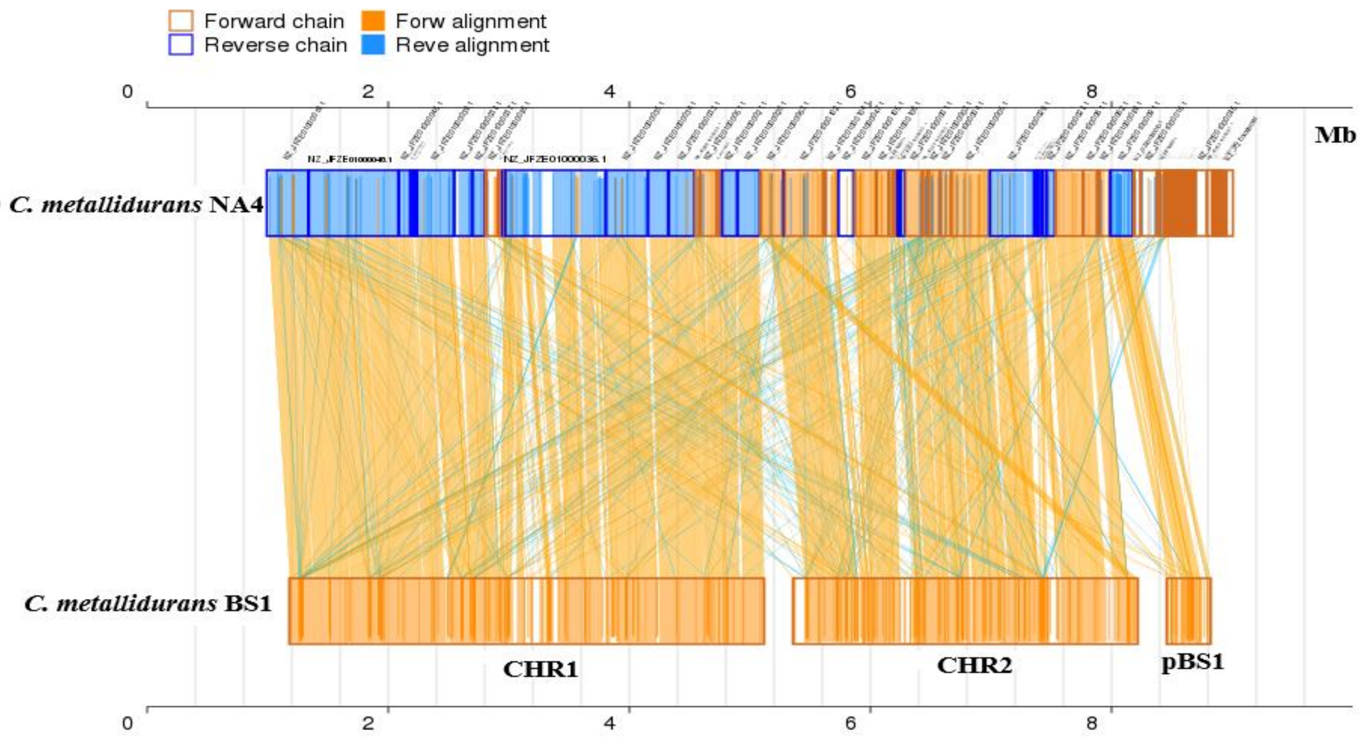

Figure_S2: Synteny plot of *C. metallidurans* strain NA4 and BS1 on nucleic acid level. Synteny was constructed using MCScanX tookit. The chromosome, chromid and plasmids of strains BS1 were compared with genome of strain NA4. Yellow box represents the forward chain and blue box represents the reverse chain within the upper and following sequence region. In the box of sequence, the yellow region represents the nucleic acid sequence in the forward chain of this genome sequence and the blue region represents the nucleic acid sequence in the reverse chain of this genome sequence. In the middle region of two sequences, the yellow line represents the forward alignment and the blue line represents the reverse complementary alignment.

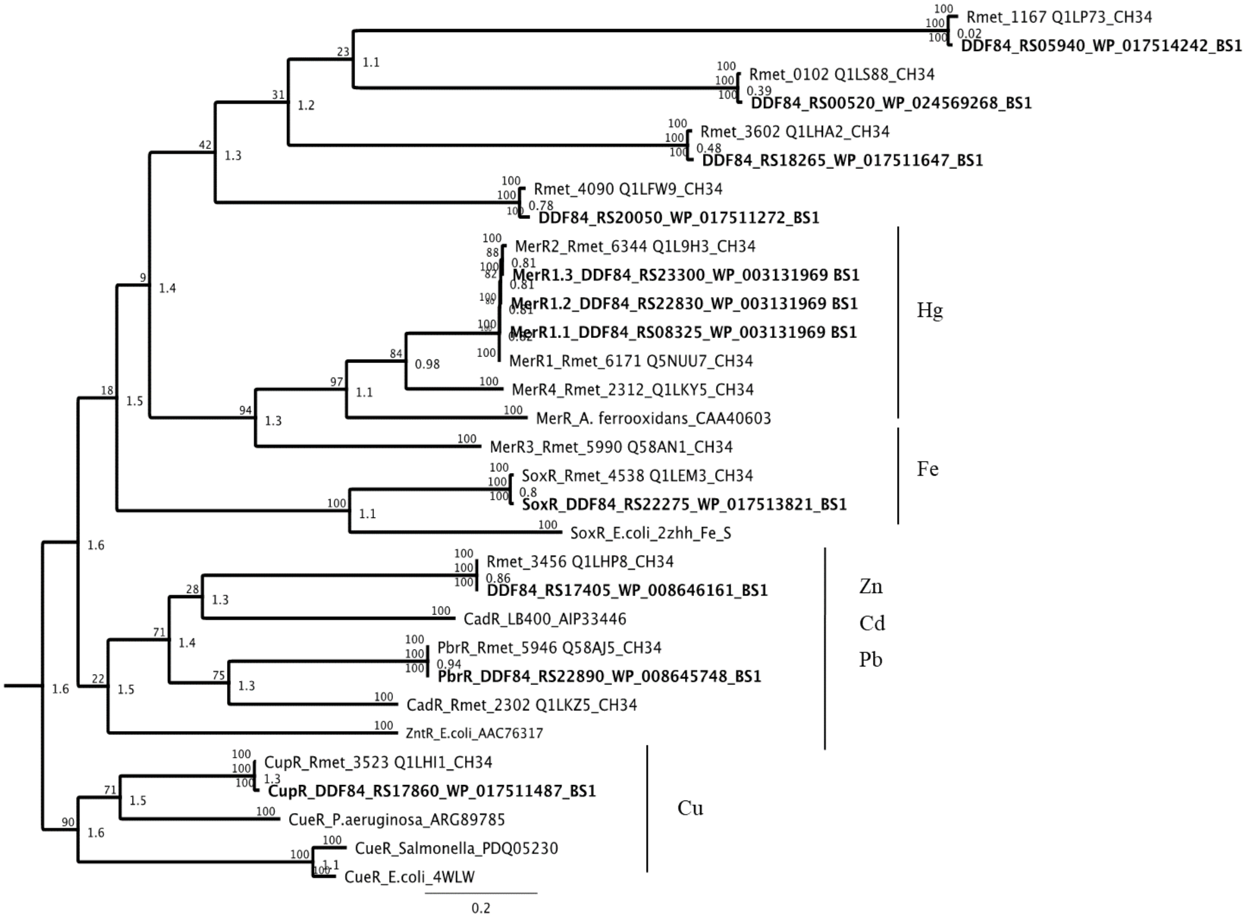

Figure S3: Molecular Phylogenetic tree based on the full amino acid sequences of the MerR members encoded on the genome of *C. metallidurans* CH34 and the homologues as well as paralogous of both The evolutionary history was inferred by using the Geneious prime 2020 0.4. (Geneious Tree Builder) (https://www.geneious.com), global alignment with free gaps, Identity (1.0/0.0), gap open penalty 12, gap extension penalty 3, 2 refinement iterations, Genetic Distance Model _ Jukes-Cantor, Neighbor-Joining, Resampling Method – Bootstrap; 100). The metals that are recognized and sensed by members of the different subfamilies are indicated (Hobman et al., 2005). The CadR$_{P.\ xenovorans\ \mathrm{LB400}}$, CueR$_{P.\ aeruginosa}$, CueR$_{E.\ coli\ \mathrm{K\text{-}12}}$, CueR$_{S.\ enterica\ subsp.\ enterica\ serovar\ Enteritidis}$, MeR$_{A.\ ferrooxidans}$ and SoxR $_{E.\ coli\ \mathrm{K\text{-}12}}$ (MeR family) amino acid sequences from *Paraburkholderia xenovorans* LB400, *Pseudomonas aeruginosa*, *Escherichia coli* strain K-12 substr. MG1655, *Salmonella enterica* subsp. enterica serovar Enteritidis and *Acidithiobacillus ferrooxidans* were used as reference (Lee, 2002; Van Houdt et al., 2009). The tree is drawn to scale, with branch lengths calculated using the average pathway method; the scale bar corresponds to the number of substitutions per site, the branch labels are consensus Support (%) and Node Heights (Kearse et al., 2012).

References:

Borremans, B., Hobman, J. L., Provoost, A., and Brown, N. L. (2001). Cloning and Functional Analysis of the pbr Lead Resistance Determinant of *Ralstonia metallidurans* CH34. *J. Bacteriol.* 183, 5651–5658. doi:10.1128/JB.183.19.5651.

Diels, L., Faelen, M., and M Mergeay (1985). Mercury transposons from plasmids governing multiple resistance to heavy-metals in *Alcaligenes eutrophus* Ch34. *ARCHIVES* 10, 312–325.

Grass, G., Grosse, C., and Nies, D. H. (2000). Regulation of the cnr cobalt and nickel resistance determinant from Ralstonia sp. strain CH34. *J. Bacteriol.* 182, 1390–1398. Available at: http://www.ncbi.nlm.nih.gov/pubmed/10671463.

Grosse, C., Anton, A., Hoffmann, T., Franke, S., Schleuder, G., and Nies, D. (2004). Identification of a regulatory pathway that controls the heavy-metal resistance system Czc via promoter czcNp in *Ralstonia metallidurans*. *Arch. Microbiol.* 182, 109–118. doi:10.1007/s00203-004-0670-8.

Grosse, C., Grass, G., Anton, A., Franke, S., Santos, A. N., Lawley, B., et al. (1999). Transcriptional organization of the czc heavy-metal homeostasis determinant from *Alcaligenes eutrophus*. *J. Bacteriol.* 181, 2385–2393. Available at: http://www.ncbi.nlm.nih.gov/entrez/query.fcgi?cmd=Retrieve&db=PubMed&dopt=Citation&list_uids=10198000.

Juhnke, S., Peitzsch, N., Hubener, N., Grosse, C., and Nies, D. H. (2002). New genes involved in chromate resistance in Ralstonia metallidurans strain CH34. *Arch. Microbiol.* 179, 15–25. doi:10.1007/s00203-002-0492-5.

Juhnke, S., Peitzsch, N., Hubener, N., Grosse, C., and Nies, D. H. (2004). New genes involved in chromate resistance in Ralstonia metallidurans strain CH34 (vol 179, pg 15, 2002). *Arch. Microbiol.* 181, 390. doi:10.1007/s00203-004-0665-5.

Kearse, M., Moir, R., Wilson, A., Stones-Havas, S., Cheung, M., Sturrock, S., et al. (2012). Geneious Basic: An integrated and extendable desktop software platform for the organization and analysis of sequence data. *Bioinformatics* 28, 1647–1649. doi:10.1093/bioinformatics/bts199.

Liesegang, H., Lemke, K., Siddiqui, R. A., and Schlegel, H. G. (1993). Characterization of the inducible nickel and cobalt resistance determinant cnr from pMOL28 of Alcaligenes eutrophus CH34. *J. Bacteriol.* 175, 767–778. doi:10.1128/jb.175.3.767-778.1993.

Mergeay, M., Monchy, S., Vallaeys, T., Auquier, V., Benotmane, A., Bertin, P., et al. (2003). *Ralstonia metallidurans* , a bacterium specifically adapted to toxic metals: towards a catalogue of metal-responsive genes. *FEMS Microbiol. Rev.* 27, 385–410. doi:10.1016/S0168-6445(03)00045-7.

Monchy, S., Benotmane, M. A., Janssen, P., Vallaeys, T., Taghavi, S., van der Lelie, D., et al. (2007). Plasmids pMOL28 and pMOL30 of *Cupriavidus metallidurans* Are Specialized in the Maximal Viable Response to Heavy Metals. *J. Bacteriol.* 189, 7417–7425. doi:10.1128/JB.00375-07.

Monchy, S., Benotmane, M. A., Wattiez, R., van Aelst, S., Auquier, V., Borremans, B., et al. (2006). Transcriptomic and proteomic analyses of the pMOL30-encoded copper resistance in Cupriavidus metallidurans strain CH34. *Microbiology* 152, 1765–1776. doi:10.1099/mic.0.28593-0.

Nies, A., Nies, D. H., and Silver, S. (1990). Nucleotide sequence and expression of a plasmid-encoded chromate resistance determinant from Alcaligenes eutrophus. *J. Biol. Chem.* 265, 5648–5653.

Nies, D. H. (2016). The biological chemistry of the transition metal "transportome" of *Cupriavidus metallidurans*. *Metallomics* 8, 481–507. doi:10.1039/C5MT00320B.

Nies, D. H., Rehbein, G., Hoffmann, T., Baumann, C., and Grosse, C. (2006). Paralogs of Genes Encoding Metal Resistance Proteins in *Cupriavidus metallidurans* Strain CH34. *J. Mol. Microbiol. Biotechnol.* 11, 82–93. doi:10.1159/000092820.

Nies, D. H., and Silver, S. (1989). Plasmid-determined inducible efflux is responsible for resistance to cadmium, zinc, and cobalt in Alcaligenes eutrophus. *J. Bacteriol.* doi:10.1128/jb.171.2.896-900.1989.

Siguier, P. (2006). ISfinder: the reference centre for bacterial insertion sequences. *Nucleic Acids Res.* 34, D32–D36. doi:10.1093/nar/gkj014.

Van Houdt, R., Monchy, S., Leys, N., and Mergeay, M. (2009). New mobile genetic elements in *Cupriavidus metallidurans* CH34, their possible roles and occurrence in other bacteria. *Antonie Van Leeuwenhoek* 96, 205–226. doi:10.1007/s10482-009-9345-4.

Wiesemann, N., Bütof, L., Herzberg, M., Hause, G., Berthold, L., Etschmann, B., et al. (2017). Synergistic Toxicity of Copper and Gold Compounds in *Cupriavidus metallidurans*. *Appl. Environ. Microbiol.* 83, 1–17. doi:10.1128/AEM.01679-17.

Wiesemann, N., Mohr, J., Grosse, C., Herzberg, M., Hause, G., Reith, F., et al. (2013). Influence of Copper Resistance Determinants on Gold Transformation by *Cupriavidus metallidurans* Strain CH34. *J. Bacteriol.* 195, 2298–2308. doi:10.1128/JB.01951-12.

Zhang, Y. B., Monchy, S., Greenberg, B., Mergeay, M., Gang, O., Taghavi, S., et al. (2009). ArsR arsenic-resistance regulatory protein from *Cupriavidus metallidurans* CH34. *Antonie van Leeuwenhoek*, 96, 161–170. doi:10.1007/s10482-009-9313-z.
